# Supplementary material for: Unzipped genome assemblies of polyploid root-knot nematodes reveal unusual and clade-specific telomeric repeats
Source: Nat Commun. 2024 Feb 5;15:773. doi: 10.1038/s41467-024-44914-y (PMC10844300; doi:10.1038/s41467-024-44914-y)
Supplement: Supplementary file 1 — Supplementary Information [file 41467_2024_44914_MOESM1_ESM.pdf]

Supplementary information

Supplementary Figure. 1: k-mer estimation of genome ploidy

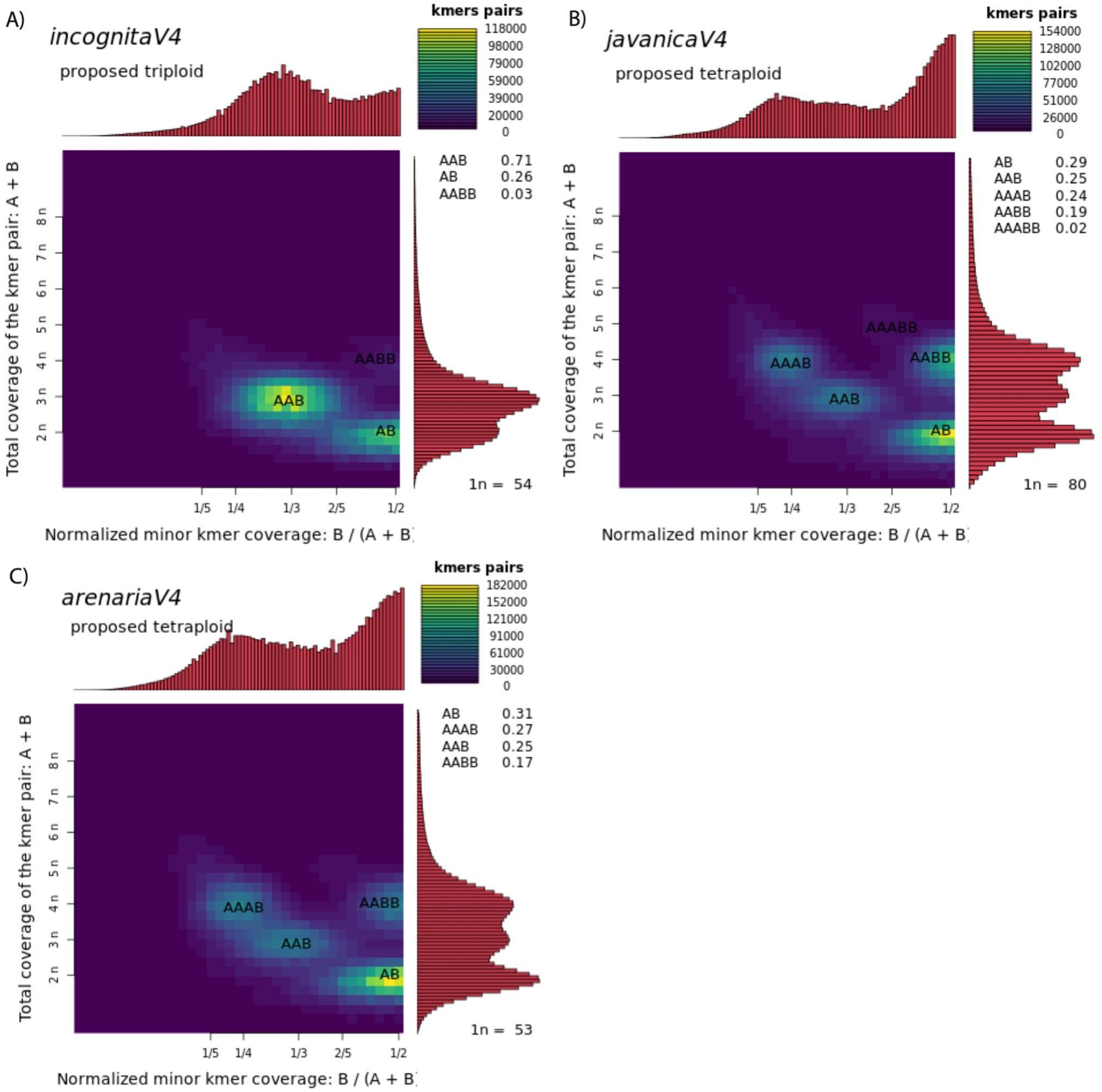

Smudgeplot[1] k-mer distribution analysis of the Illumina reads used for polishing the contigs predicted a triploid (3n 'AAB') genome structure for *M. incognita* (**A**) and tetraploid (4n 'AABB') genome structures for both *M. javanica* (**B**) and *M. arenearia* (**C**).

Supplementary Figure. 2: k-mer estimation of genome size and heterozygosity

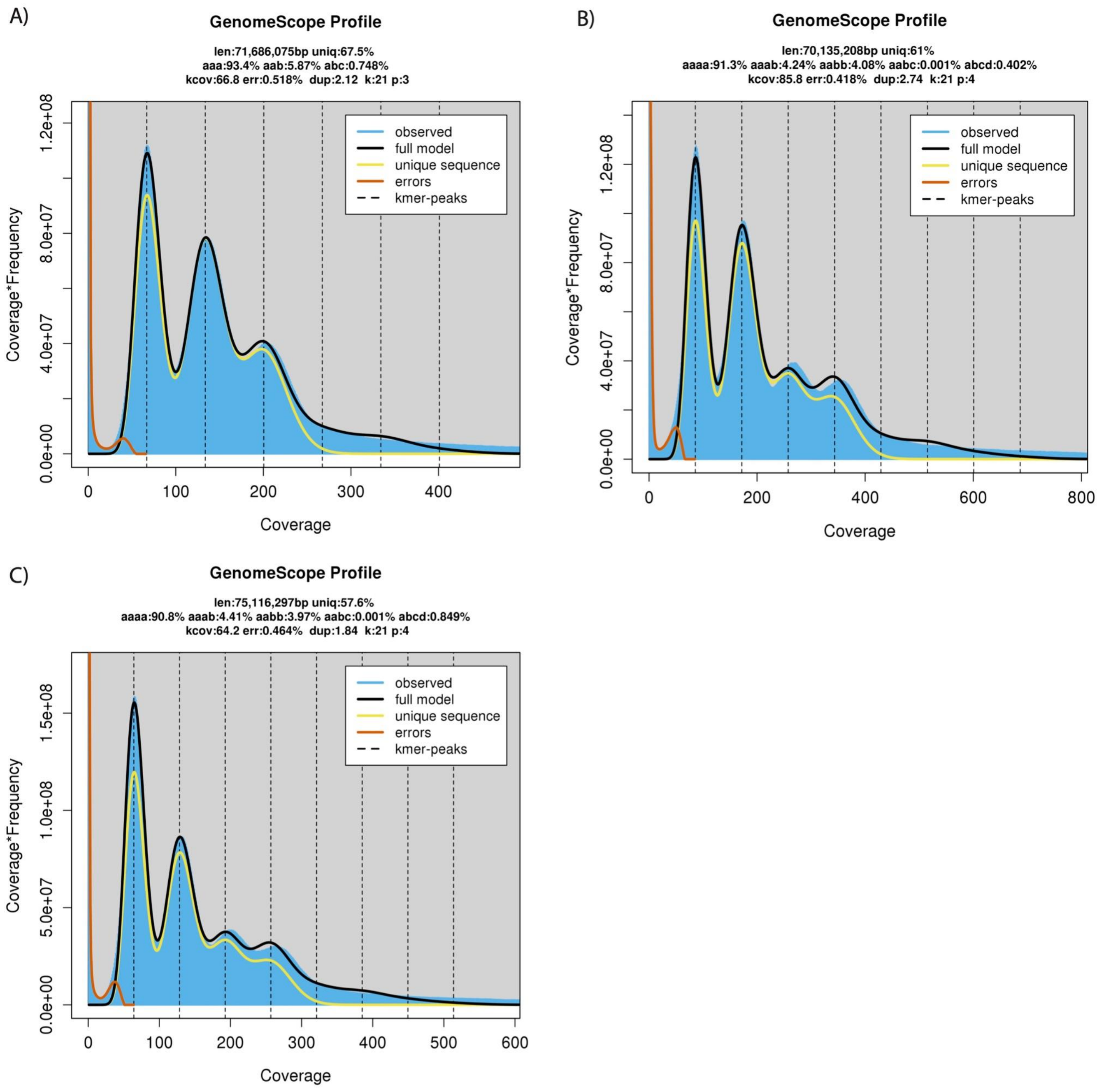

Estimation of genome size and nucleotide divergence between homoeologous genome copies using GenomeScope2[1]. (**A**) Setting the ploidy at 3n in *M. incognita* yielded an estimated haploid genome size of ca. 71.69Mb (and thus a total genome size of ca 215Mb), with an average nucleotide divergence between homoeologous genome copies of 6.6%. (**B**) Setting the ploidy at 4n in *M. javanica* yielded an estimated haploid genome size of ca. 70.14 Mb (and total genome size of 280.6 Mb) with an average nucleotide divergence between genome copies of 8.7%. (**C**) Setting the ploidy at 4n in *M. arenaria* yielded an estimated haploid genome size of ca. 75.12 Mb (total genome size of ca. 300.5 Mb) with an average nucleotide divergence between genome copies of 9.2%.

Supplementary Figure 3 k-mer estimation of genome assembly completeness

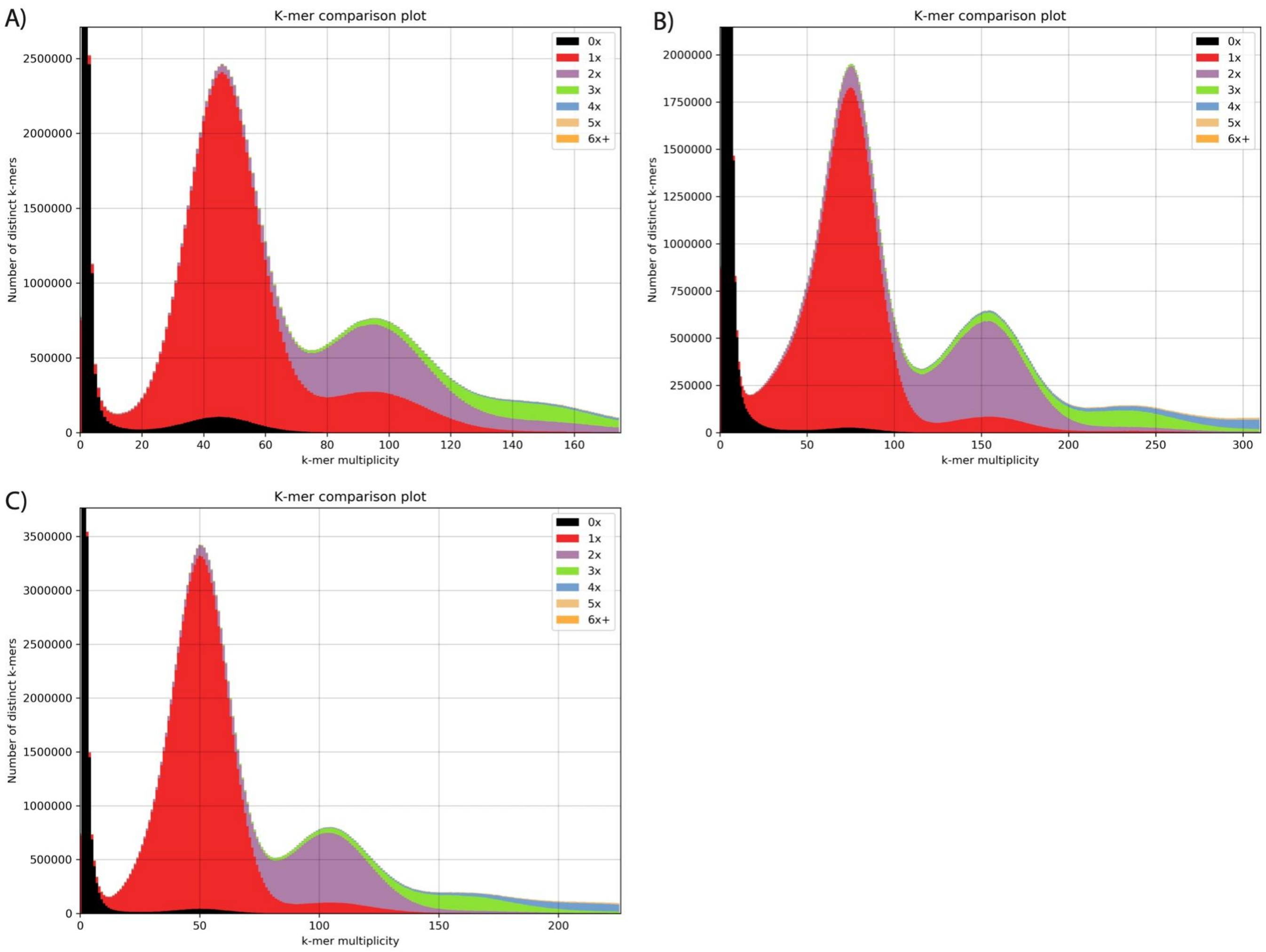

KAT[2] comparative analysis of the distribution of Illumina reads k-mers in the *M. incognita* (**A**), *M. javanica* (**B**), and *M. arenaria* (**C**) genome assemblies. Very few k-mers in the reads were not represented in the assembly (0X black curve), suggesting almost all the information present in the reads was retrieved in the assemblies. This observation and the congruence between genome assembly sizes and those estimated via flow cytometry[3] suggest complete genomes with homoeologous genome copies having been mostly unzipped during assembly.

Supplementary Figure 4: genome contamination assessment with blobtools

1) *M. incognita*

A)

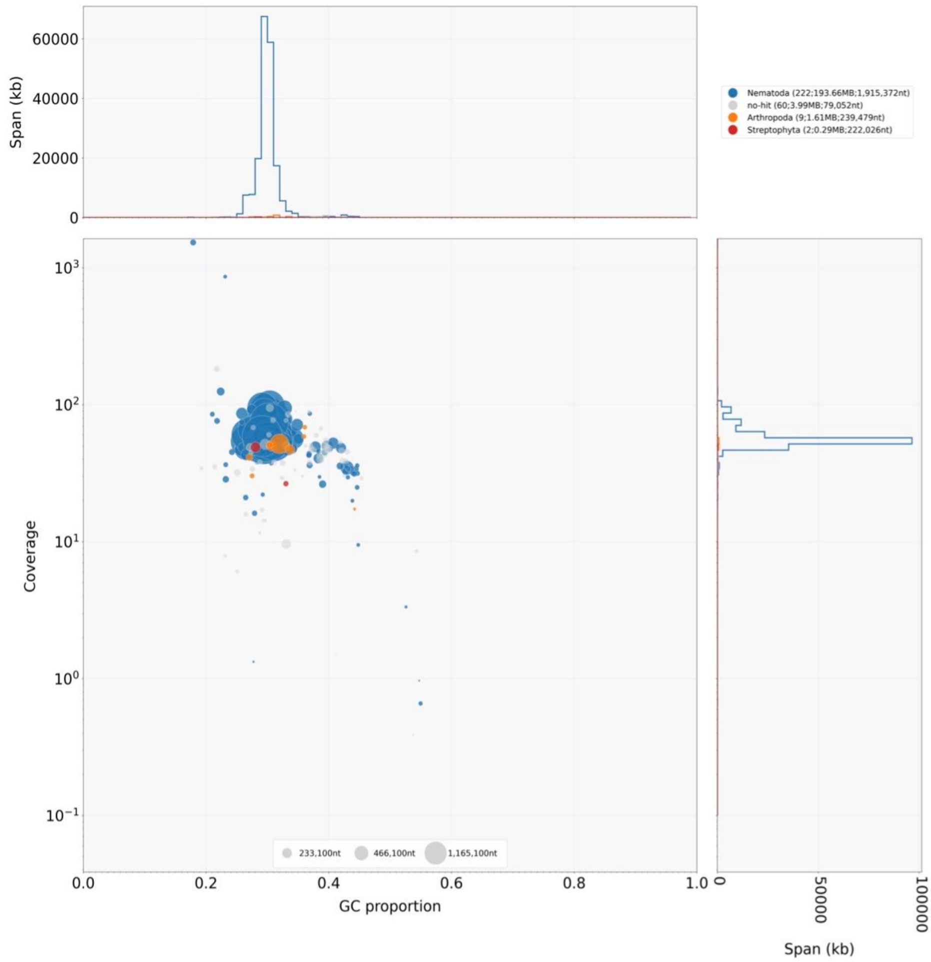

B)

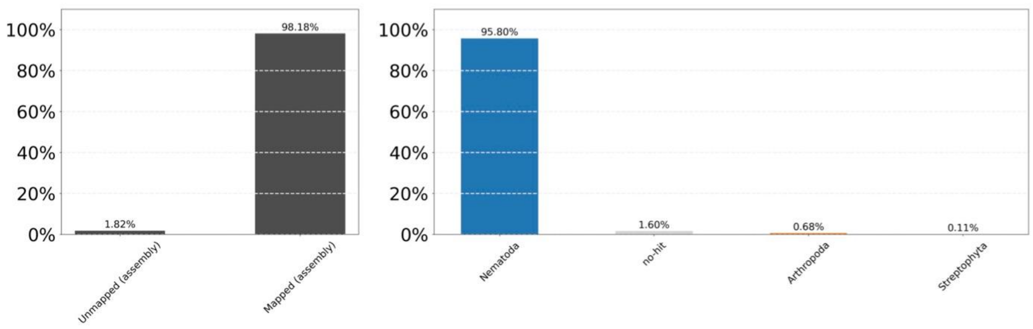

No evident trace of contamination could be identified. Two contigs with very high coverage corresponded to the mitochondrial genome of *M. incognita* and were removed from the final nuclear genome assembly.

2) *M. javanica*

A)

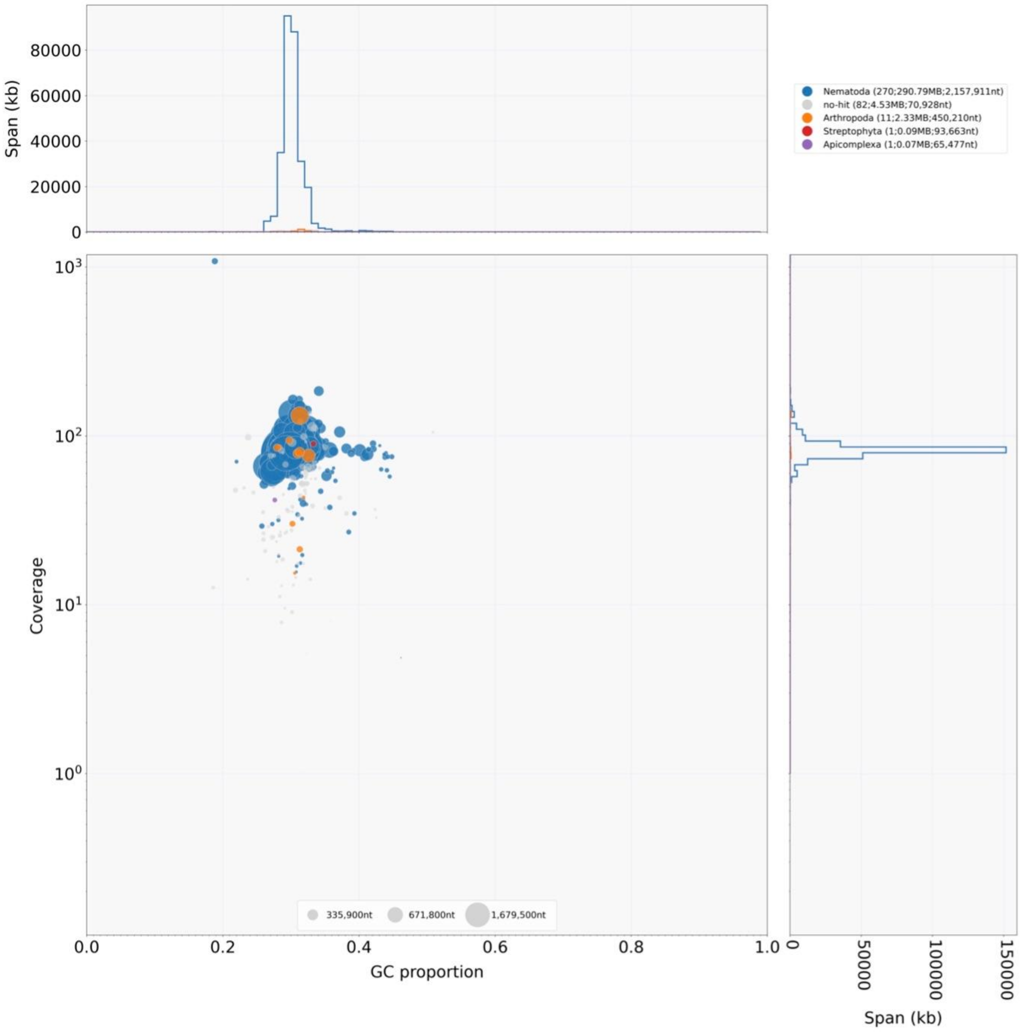

B)

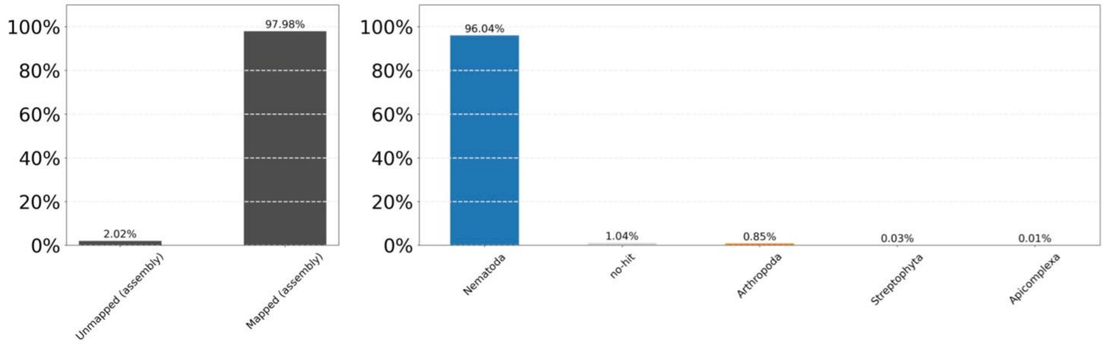

The majority of the contigs had approximately the same coverage, and one contig identified with a high coverage was annotated as the mitochondrial genome of *M. javanica* and removed from the assembly. A low percentage of the contigs was mapped as Arthropoda (0.85%), but this can be the result of a lack of information on the NCBI database.

3) *M. arenaria*

A)

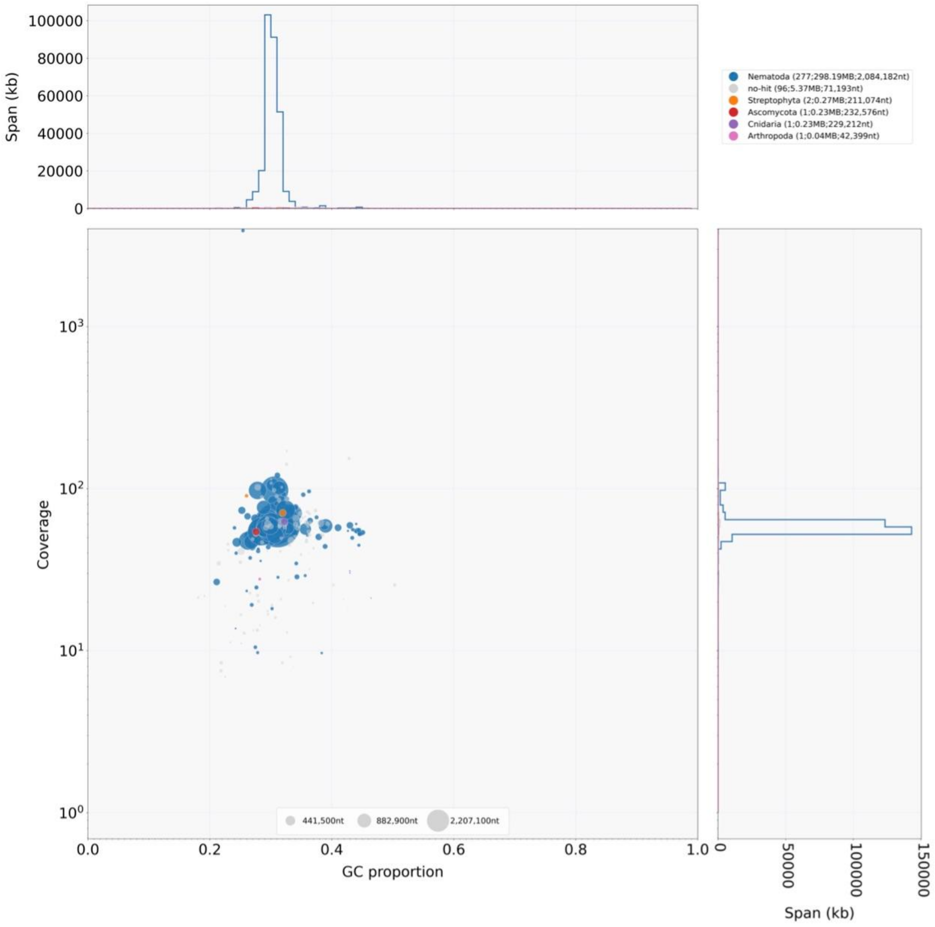

B

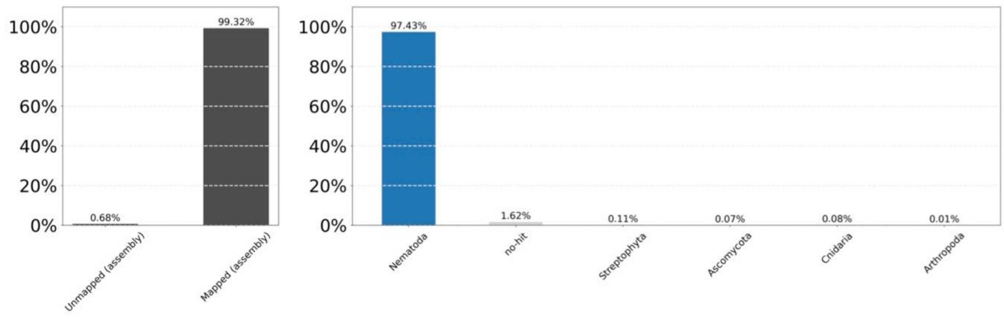

One contig was identified with a high coverage (> 10^3) compared to the others. This contig corresponded to the mitochondrial genome of *M. arenaria* and was eliminated from the final assembly. No contamination was observed in the final assembly.

Supplementary Table 1: Statistics of EuGene predictions

Feature / species	*Minc*	*Mjav*	*Mare*	*Mluci*
Predicted genes	54,816	64,689	63,434	52,935
Protein-coding	52,182	61,060	59,500	49,988
Avg. intergenic distance (coding genes) in bp	1,442.7	2,563.3	2,745.6	1,685.4
Avg. length protein-coding genes (bp)	2,254	2,102	2,122	2,333
Percent of the genome coding	23.6%	19.2%	18.5%	22.3%
GC% in coding regions	34.43%	34.63%	34.63%	34.56%
Percent of genes with introns	75%	82%	83%	79%
Avg. introns / gene	4.4	4.7	4.8	4.7
Avg. intron length	216.4	203.8	203.1	230.6
Percent of noncanonical (GC) splice donor	1.2%	1.0%	1.0%	1.3%
Non protein-coding genes	2,634	3,692	3,934	2,947
GC% of non-coding genes	38.6%	39.97%	40.9%	38.3%
Percent of genome masked by RED[1]	15.8%	23.8%	24.3%	16.4%

1: regions masked by EuGene[4] using RED[5] before gene prediction because they are repetitive with no evidence for transcription.

Supplementary Figure 5: Subgenome assignment based on gene collinearity and Ks.

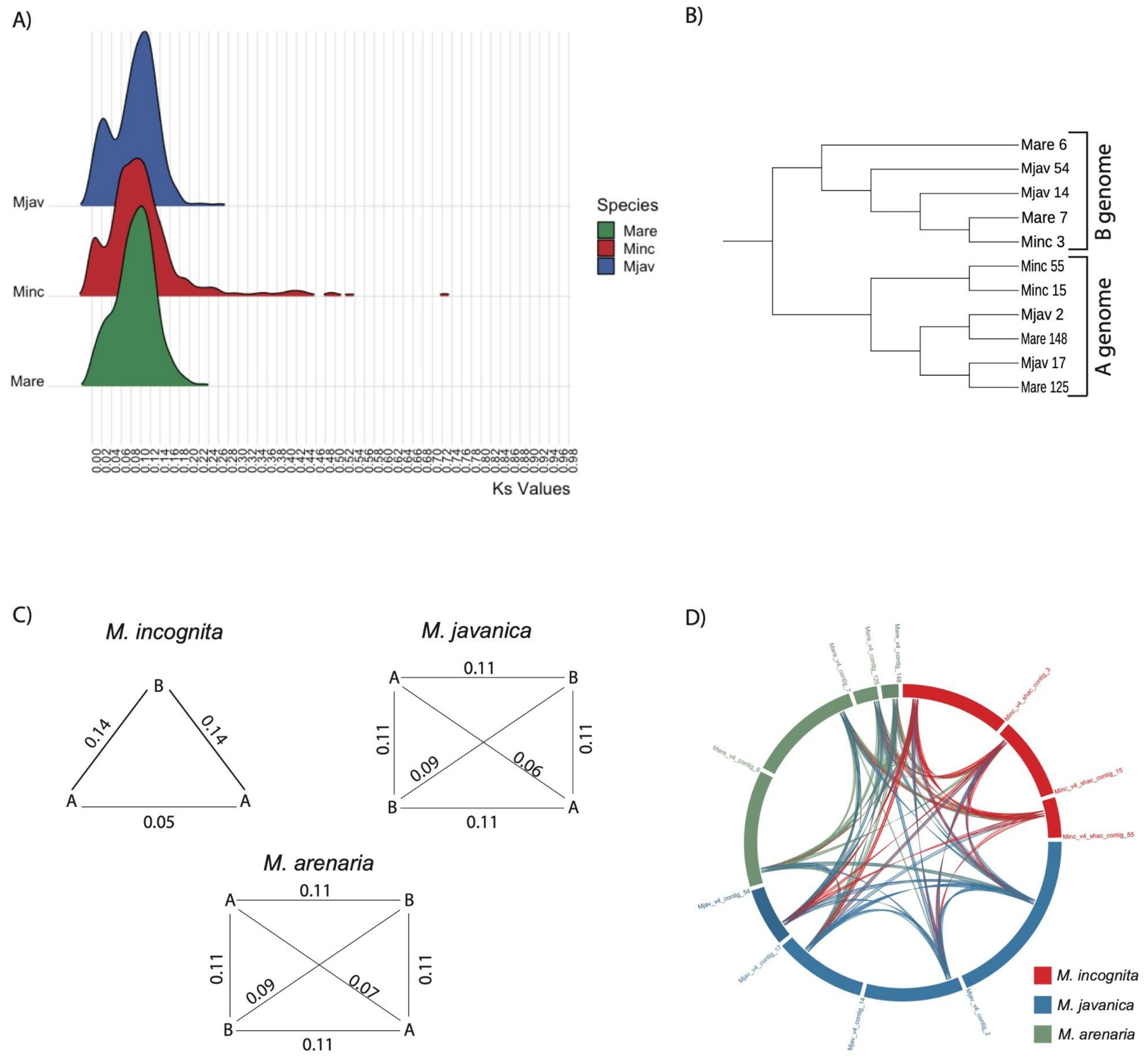

A) Distribution of median Ks values between genes constituting triplicated (*Minc*) and 'quadruplicated' (*Mjav*, *Mare*) blocks, showing two peaks. In *Minc*, the lower Ks value represents the divergence between A-A' while the higher value represents the divergence between A-B. This property allowed determining which *Minc* contigs were A and which were B. Similarly, the lower Ks value represents the divergence between A-A' or B-B' while the higher value represents the divergence between As and Bs genomes in *Mjav* and *Mare*. **B)** Example of a topology obtained by computing a Euclidean distance based on the median Ks between conserved collinear blocks of *Minc*, *Mjav,* and *Mare*. Based on the position of A and B *Minc* contigs in the tree, the contigs of *Mjav* and *Mare* were assigned to either an A or B subgenome. **C)** Median Ks values for *Minc*, *Mare*, and *Mjav* sub-genome comparisons. **D)** Example of collinear blocks of at least 10 genes conserved between the 3 species and present in 3 copies in *Minc* while in 4 copies in *Mjav* and *Mare*, in total we could find 110 such conserved collinear blocks.

Supplementary Figure 6: telomeric repeat ancestral state reconstruction

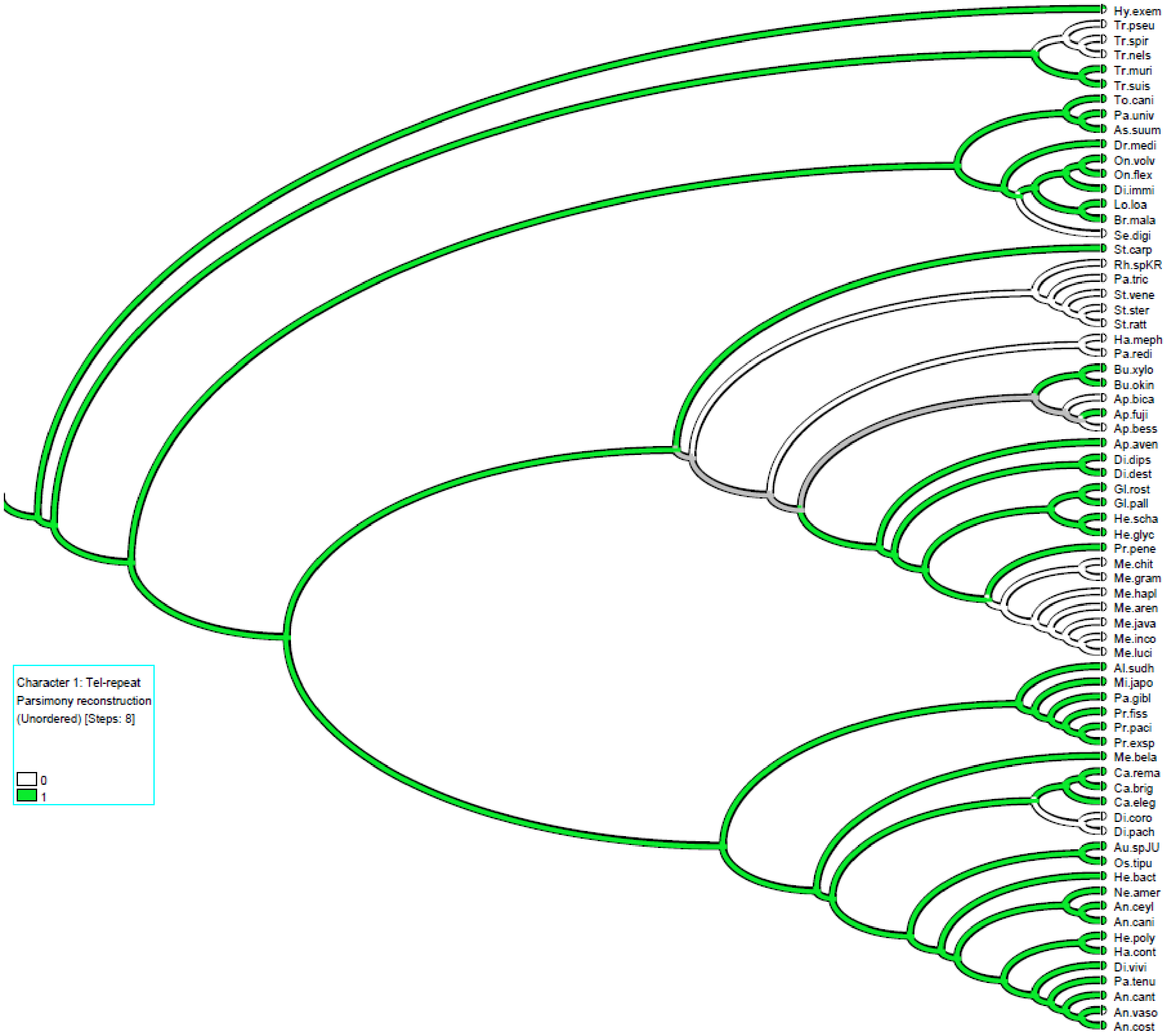

Based on presence / absence of the (TTAGGC)n telomeric repeat of *C. elegans* in nematode genomes, ancestral states were reconstructed using parsimony in Mesquite[6]. Green means (TTAGGC)n present and white absent, gray means presence and absence are equi-parsimonious. In the tardigrade *Hypsibus dujardini*, another simple repeat, (GATGGGTTTT)n, was described as a candidate telomeric repeat[7].

Supplementary Figure 7: telomerase ancestral state reconstruction

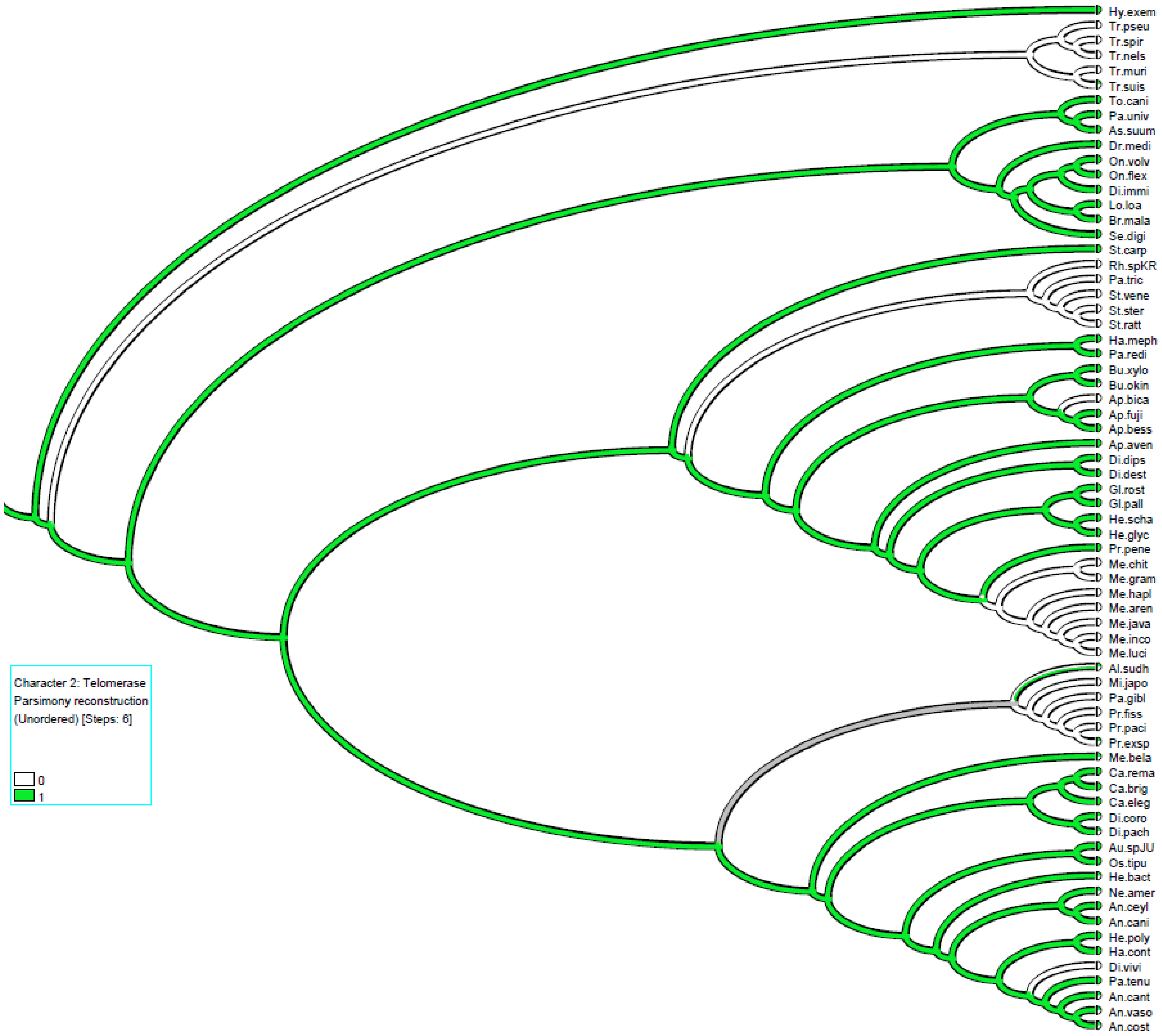

Based on presence / absence of the telomerase enzyme in nematode proteomes or telomerase gene in the genomes, ancestral states were reconstructed using parsimony in Mesquite[6]. Green means telomerase present and white absent.

Supplementary Figure 8: enriched motif at *M. incognita* contig ends

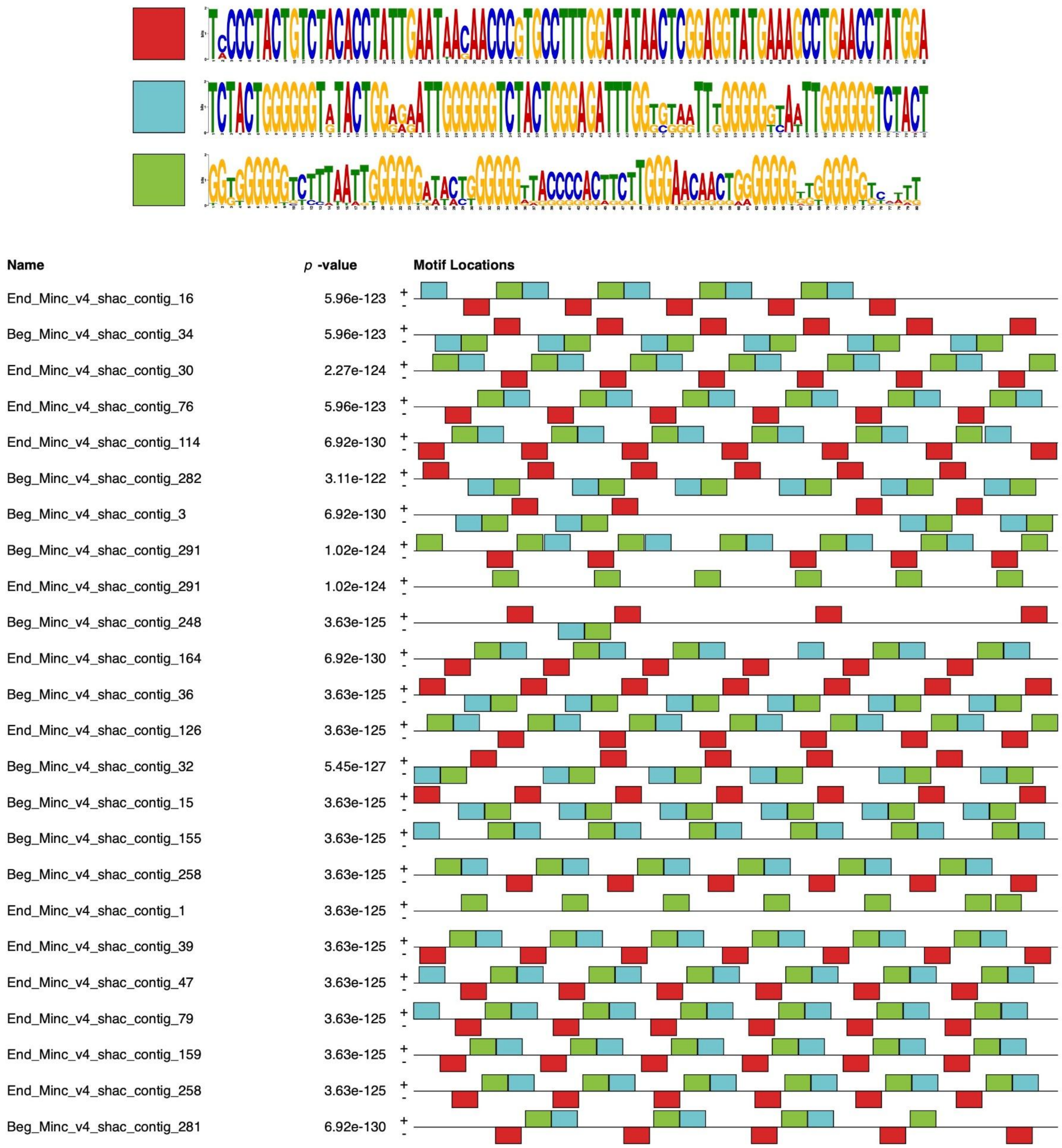

Enriched repeated motifs by increasing p-value and their distribution on contig extremities (first and last 2kb) in the *M. incognita* genome. Red: *Minc* motif-1 (MiM1), blue motif-2 (MiM2), and green: motif-3 (MiM3).

Supplementary Figure 9: enriched motif at *M. javanica* contig ends

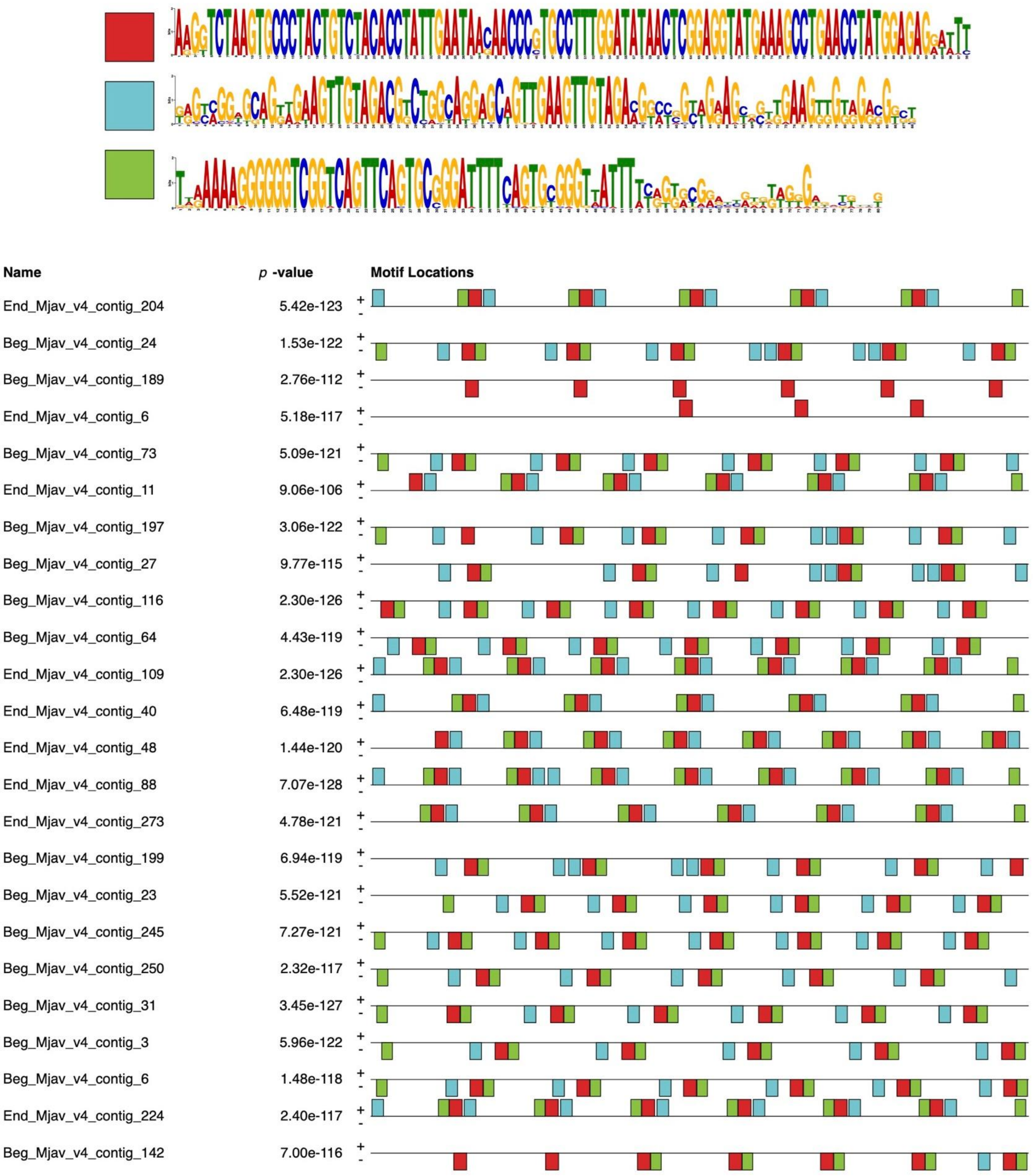

Enriched repeated motifs by increasing p-value and their distribution on contig extremities (first and last 5kb) in the *M. javanica* genome. Red: *Mjav* motif-1 (MjM1), blue motif-2 (MjM2), and green: motif-3 (MjM3).

Supplementary Figure 10: enriched motif at *M. arenaria* contig ends

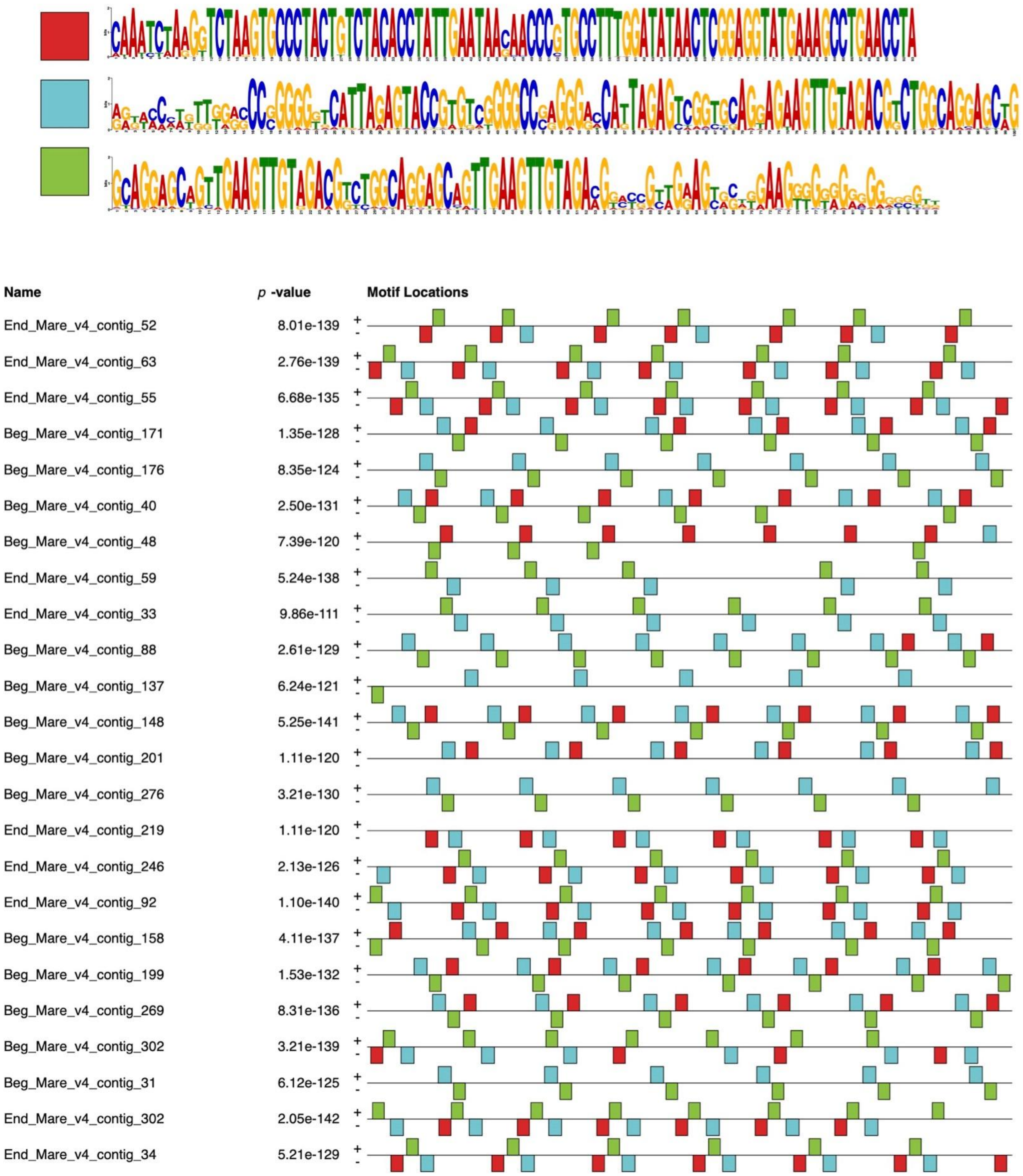

Enriched repeated motifs by increasing p-value and their distribution on contig extremities (first and last 5kb) in the *M. arenaria* genome. Red: *Mare* motif-1 (MaM1), blue motif-2 (MaM2), and green: motif-3 (MaM3).

Supplementary Figure 11: consensus of the enriched repeat at *M. incognita* contig extremities

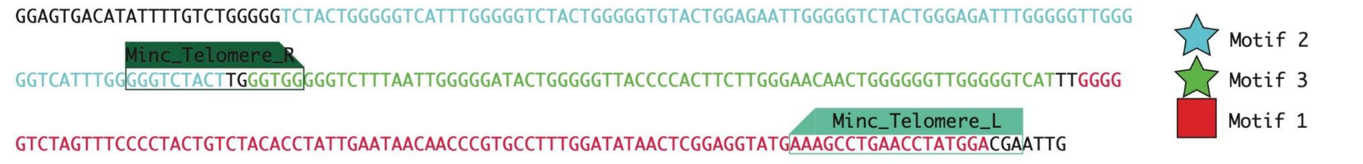

Positions of the primers used for PCR and FISH experiments are indicated as Minc_Telomere-R and Minc_Telomere_L

GGAGTGACATATTTTGTCTGGGGGTCTACTGGGGGTCATTTGGGGGTCTACTGGGGGTGTACTGGAGAATT
GGGGGTCTACTGGGAGATTTGGGGGTTGGGGGTCATTTGG*GGGTCTACTTGGGTGGG*GGTCTTTAATTGG
GGGATACTGGGGGTTACCCCACTTCTTGGGAACAACTGGGGGGTTGGGGGTCATTTGGGGGTCTAGTTTC
CCCTACTGTCTACACCTATTGAATAACAACCCGTGCCTTTGGATATAACTCGGAGGTATG*AAAGCCTGAAC*
CTATGGACGAATTG

(in italics: positions of the primers)

Multiple sequence alignment of the repeated unit made of motif-2 (blue), motif-3 (green), and motif-1 (red) as defined in supplementary Figure 7 allowed deducing a ca. 250 - 300 bp consensus sequence.

Supplementary Figure 12: consensus of the enriched repeat at *M. javanica* contigs extremities

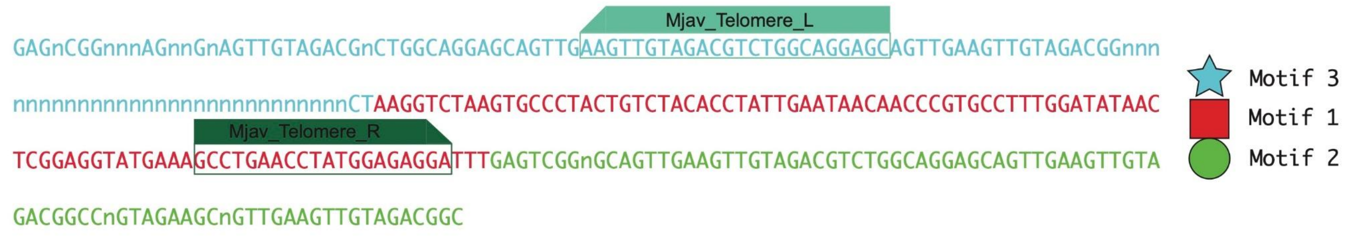

GAGnCGGnnnAGnnGnAGTTGTAGACGnCTGGCAGGAGCAGTTG*AAGTTGTAGACGTCTGGCAGG*AGCAG
TTGAAGTTGTAGACGGnnnnnnnnnnnnnnnnnnnnnnnnnnnnnCTAAGGTCTAAGTGCCCTACTGTCTACAC
CTATTGAATAACAACCCGTGCCTTTGGATATAACTCGGAGGTATGAAA*GCCTGAACCTATGGAGAGGA*TTT
TnAAAAAGGGGGGTCGGTCAGTTCAGTGCGGGAnTTTTCAGTGCGGGTTATTTTCGnnTGnnGGACGAATT
GGGGAGTGAnATnnnn

(in italics: positions of the primers)

Supplementary Figure 13: consensus of the enriched repeat at *M. arenaria* contig extremities

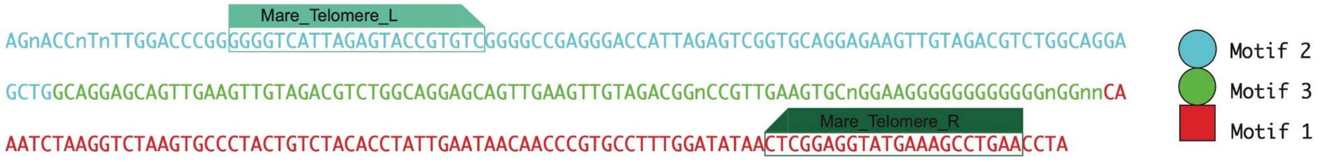

AGnACCnTnTTGGACCCGG*GGGGTCATTAGAGTACCGTGTC*GGGGCCGAGGGACCATTAGAGTCGGTGCAGGAGAAGTTGTAGACGTCTGGCAGGAGCTGGCAGGAGCAGTTGAAGTTGTAGACGTCTGGCAGGAGCAGTTGAAGTTGTAGACGGnCCGTTGAAGTGCnGGAAGGGGGGGGGGGGnGGnnCAAATCTAAGGTCTAAGTGCCCTACTGTCTACACCTATTGAATAACAACCCGTGCCTTTGGATATAA*CTCGGAGGTATGAAAGCCTGAAC*CTA

(in italics: positions of the primers)

Supplementary Figure 14: distribution of repeat patterns and G4-quadruplex on *M. incognita* contig 50kb extremities

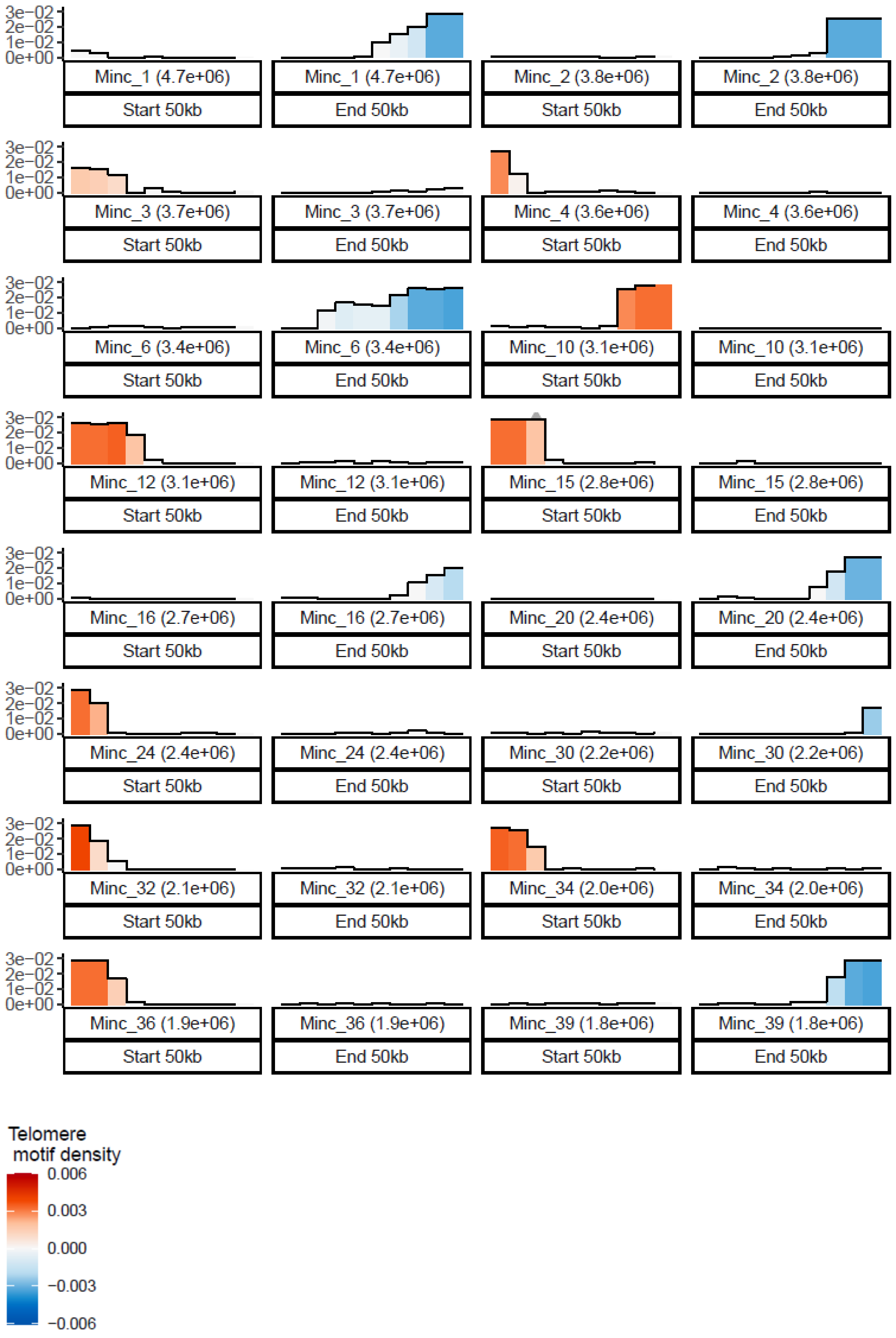

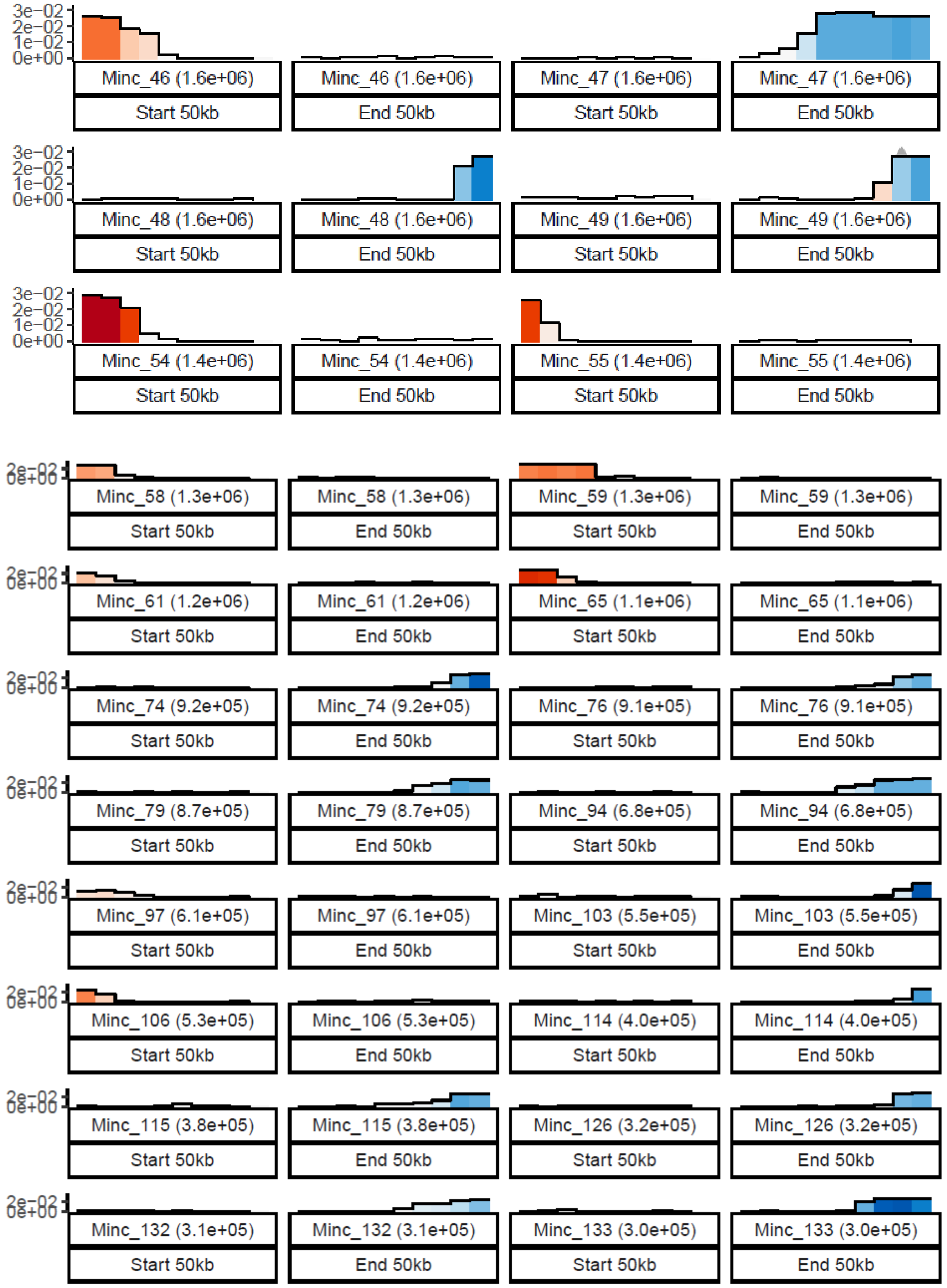
3e−02
2e−02
1e−02
0e+00
Minc_46 (1.6e+06) Start 50kb
Minc_46 (1.6e+06) End 50kb
Minc_47 (1.6e+06) Start 50kb
Minc_47 (1.6e+06) End 50kb
Minc_48 (1.6e+06) Start 50kb
Minc_48 (1.6e+06) End 50kb
Minc_49 (1.6e+06) Start 50kb
Minc_49 (1.6e+06) End 50kb
Minc_54 (1.4e+06) Start 50kb
Minc_54 (1.4e+06) End 50kb
Minc_55 (1.4e+06) Start 50kb
Minc_55 (1.4e+06) End 50kb
Minc_58 (1.3e+06) Start 50kb
Minc_58 (1.3e+06) End 50kb
Minc_59 (1.3e+06) Start 50kb
Minc_59 (1.3e+06) End 50kb
Minc_61 (1.2e+06) Start 50kb
Minc_61 (1.2e+06) End 50kb
Minc_65 (1.1e+06) Start 50kb
Minc_65 (1.1e+06) End 50kb
Minc_74 (9.2e+05) Start 50kb
Minc_74 (9.2e+05) End 50kb
Minc_76 (9.1e+05) Start 50kb
Minc_76 (9.1e+05) End 50kb
Minc_79 (8.7e+05) Start 50kb
Minc_79 (8.7e+05) End 50kb
Minc_94 (6.8e+05) Start 50kb
Minc_94 (6.8e+05) End 50kb
Minc_97 (6.1e+05) Start 50kb
Minc_97 (6.1e+05) End 50kb
Minc_103 (5.5e+05) Start 50kb
Minc_103 (5.5e+05) End 50kb
Minc_106 (5.3e+05) Start 50kb
Minc_106 (5.3e+05) End 50kb
Minc_114 (4.0e+05) Start 50kb
Minc_114 (4.0e+05) End 50kb
Minc_115 (3.8e+05) Start 50kb
Minc_115 (3.8e+05) End 50kb
Minc_126 (3.2e+05) Start 50kb
Minc_126 (3.2e+05) End 50kb
Minc_132 (3.1e+05) Start 50kb
Minc_132 (3.1e+05) End 50kb
Minc_133 (3.0e+05) Start 50kb
Minc_133 (3.0e+05) End 50kb

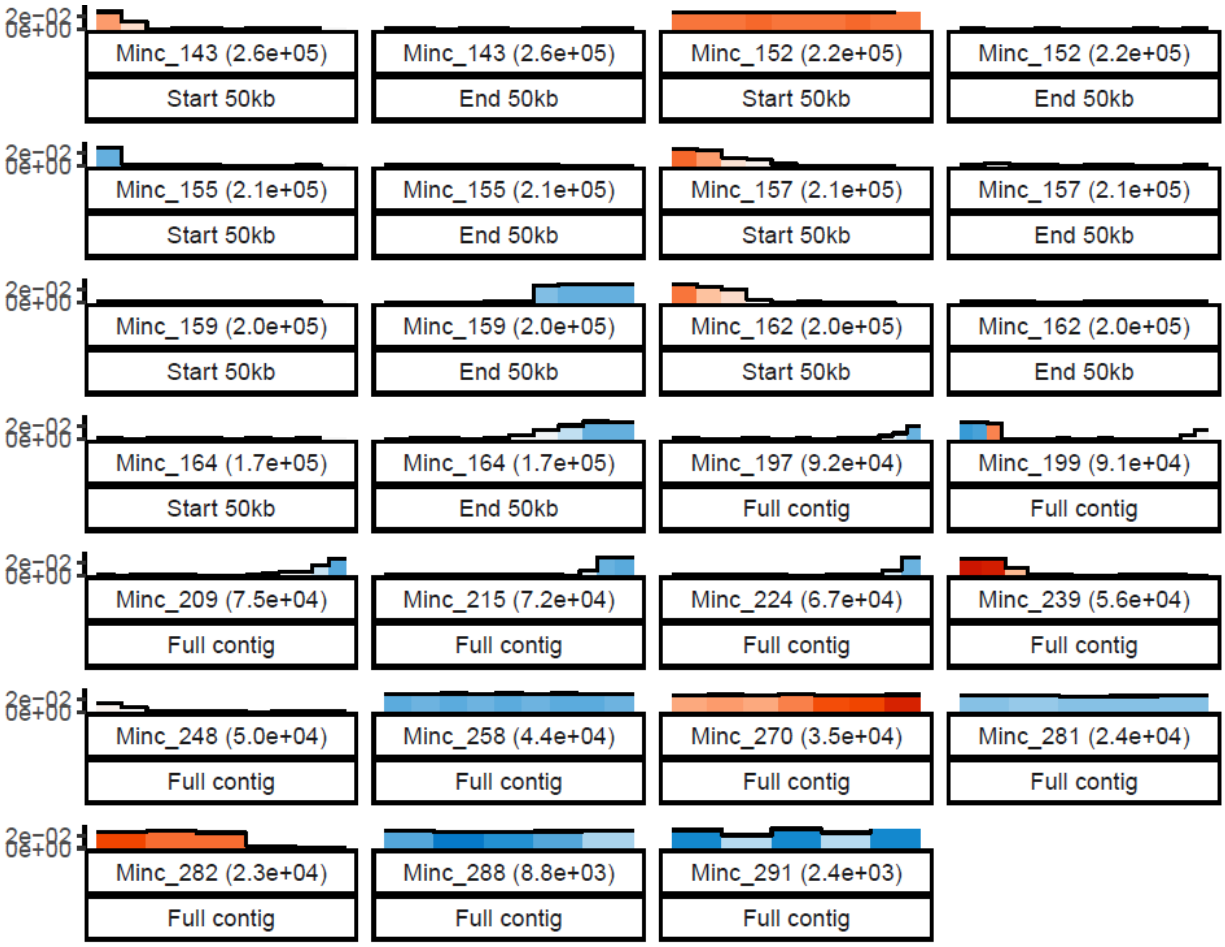

Density of repeat patterns and G4-quadruplex along *M. incognita* contig extremities. The density of *Minc* repeat units is represented by a color gradient with positive values (red) indicating a density in the sense strand and negative values (blue) indicating a density on the reverse complement strand. Gray triangles above bars indicate regions of the contigs where less than 100% of the repeat patterns are on the same strand. The heights of bars in the histogram represent the density of G4-quadruplex forming regions. The first and last 50kb of each contig containing at least 3 repeats are represented and the values are per 5kb windows on the genome. As a complement to Figure 3, a supplementary figure online represents the distribution of Minc repeats and G4s on the whole contig length in 100kb windows (https://doi.org/10.57745/1WDPE4). Source data to produce this figure is provided as a Source Data file.

Supplementary Figure 15: Distribution of repeat patterns and G4-quadruplex on *M. javanica* 50kb contig extremities

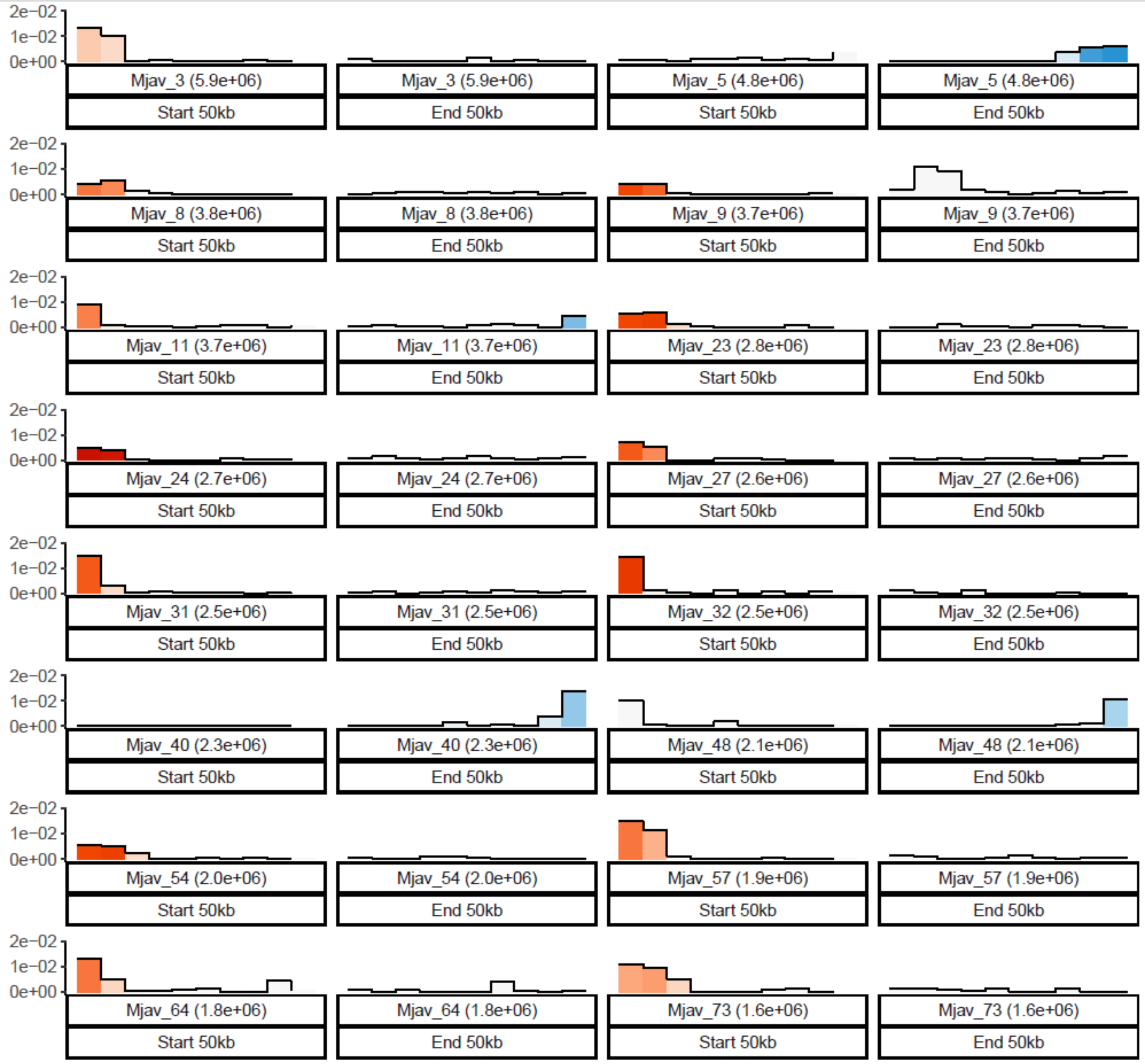

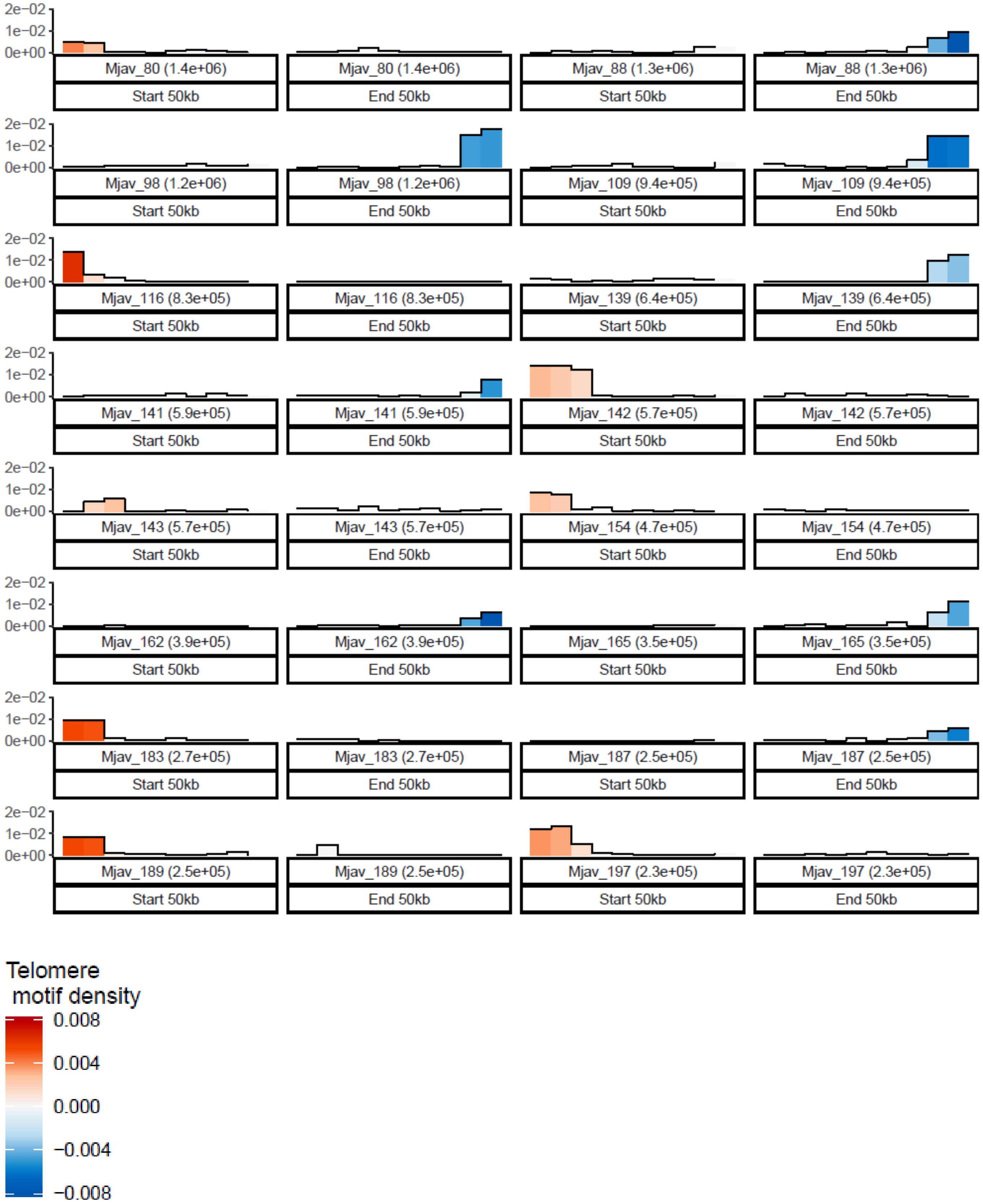
2e−02
1e−02
0e+00
Mjav_80 (1.4e+06)
Start 50kb
Mjav_80 (1.4e+06)
End 50kb
Mjav_88 (1.3e+06)
Start 50kb
Mjav_88 (1.3e+06)
End 50kb
Mjav_98 (1.2e+06)
Start 50kb
Mjav_98 (1.2e+06)
End 50kb
Mjav_109 (9.4e+05)
Start 50kb
Mjav_109 (9.4e+05)
End 50kb
Mjav_116 (8.3e+05)
Start 50kb
Mjav_116 (8.3e+05)
End 50kb
Mjav_139 (6.4e+05)
Start 50kb
Mjav_139 (6.4e+05)
End 50kb
Mjav_141 (5.9e+05)
Start 50kb
Mjav_141 (5.9e+05)
End 50kb
Mjav_142 (5.7e+05)
Start 50kb
Mjav_142 (5.7e+05)
End 50kb
Mjav_143 (5.7e+05)
Start 50kb
Mjav_143 (5.7e+05)
End 50kb
Mjav_154 (4.7e+05)
Start 50kb
Mjav_154 (4.7e+05)
End 50kb
Mjav_162 (3.9e+05)
Start 50kb
Mjav_162 (3.9e+05)
End 50kb
Mjav_165 (3.5e+05)
Start 50kb
Mjav_165 (3.5e+05)
End 50kb
Mjav_183 (2.7e+05)
Start 50kb
Mjav_183 (2.7e+05)
End 50kb
Mjav_187 (2.5e+05)
Start 50kb
Mjav_187 (2.5e+05)
End 50kb
Mjav_189 (2.5e+05)
Start 50kb
Mjav_189 (2.5e+05)
End 50kb
Mjav_197 (2.3e+05)
Start 50kb
Mjav_197 (2.3e+05)
End 50kb
Telomere motif density
0.008
0.004
0.000
−0.004
−0.008

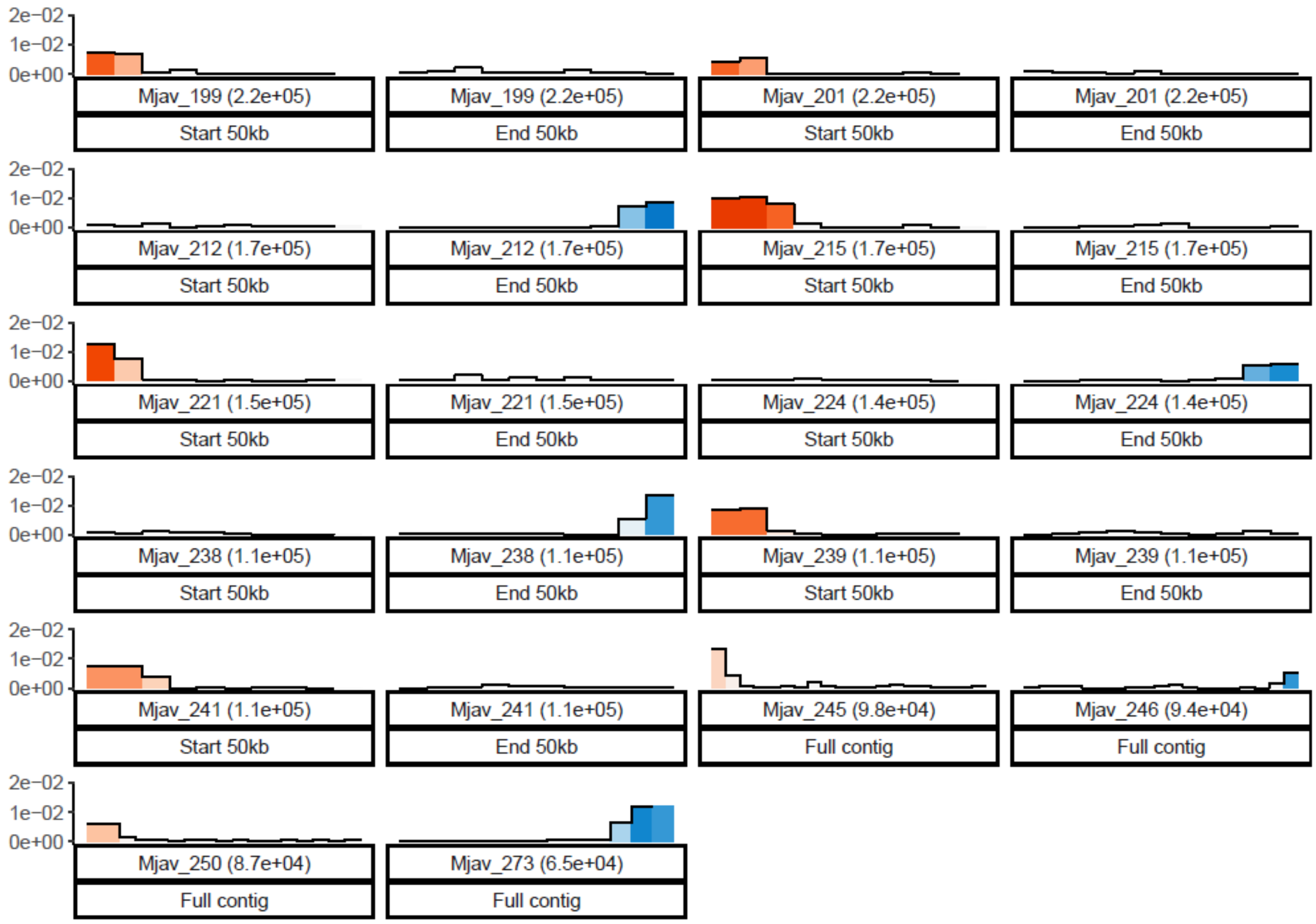

B) Density of repeat patterns and G4-quadruplex along *M. javanica* contig extremities. The density of *Mjav* repeat units is represented by a color gradient with positive values (red) indicating a density in the sense strand and negative values (blue) indicating a density on the reverse complement strand. Gray triangles above bars indicate regions of the contigs where less than 100% of the repeat patterns are on the same strand. The heights of bars in the histogram represent the density of G4-quadruplex forming regions. The first and last 50kb of each contig containing at least 3 repeats are represented and the values are per 5kb windows on the genome, contigs that do not contain repeats in their first or last 50kb are ignored. The distribution of Mjav repeats and G4s on the whole contig length in 100kb windows is available online at (https://doi.org/10.57745/EIKAQ4). Source data to produce this figure is provided as a Source Data file.

Supplementary Figure 16: Distribution of repeat patterns and G4-quadruplex on *M. arenaria* 50kb contig extremities

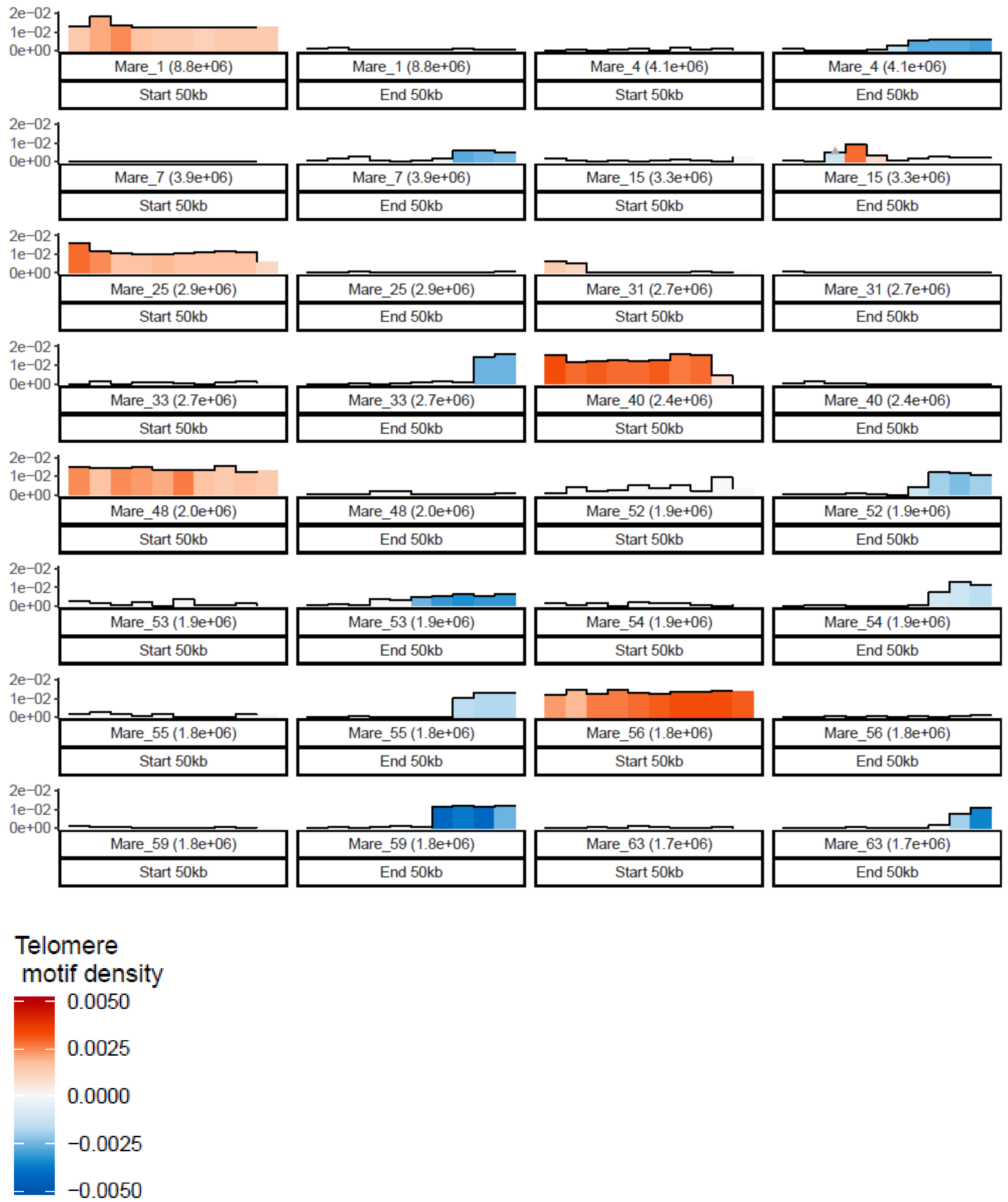

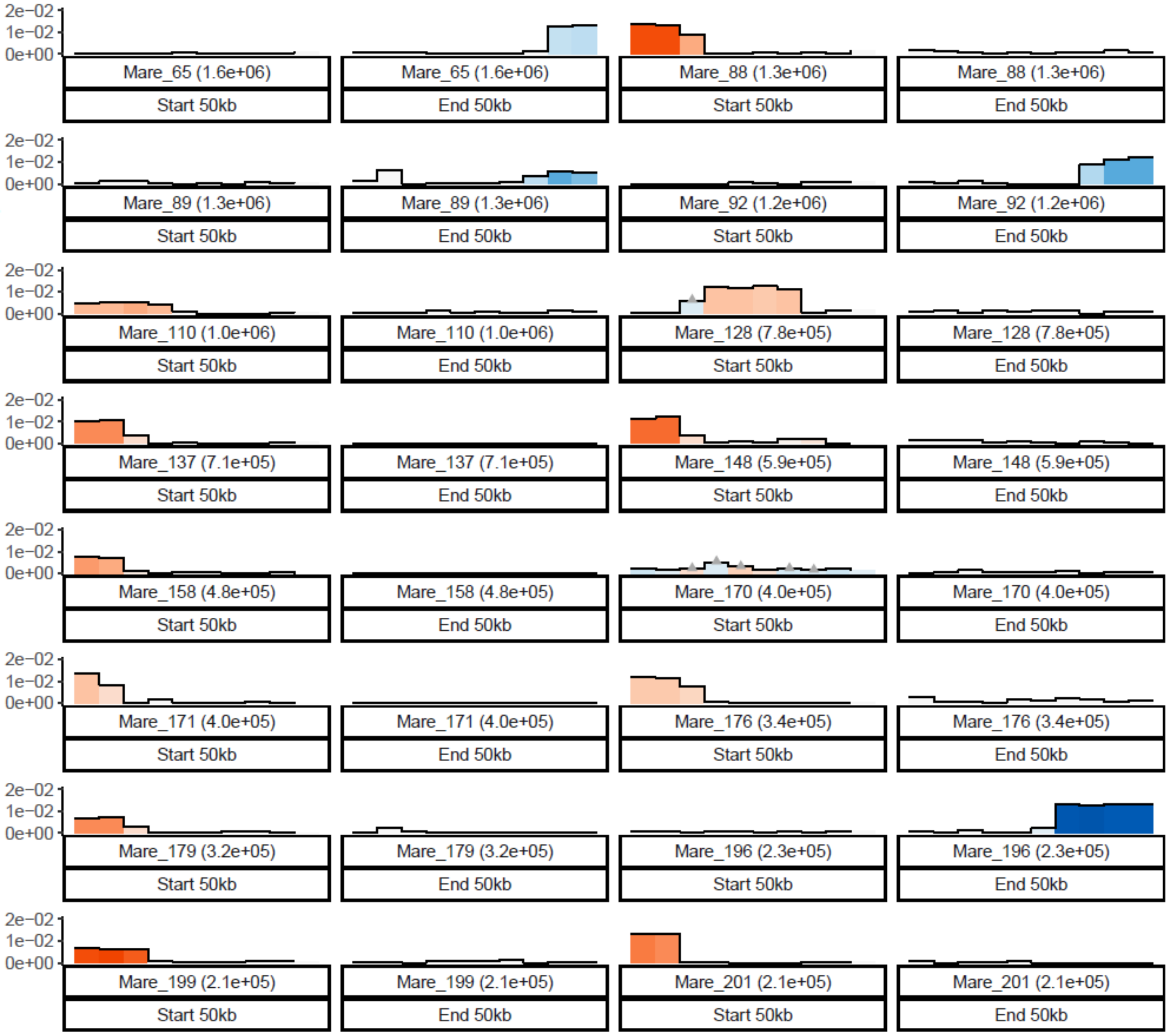
2e−02
1e−02
0e+00
Mare_65 (1.6e+06)
Start 50kb
Mare_65 (1.6e+06)
End 50kb
Mare_88 (1.3e+06)
Start 50kb
Mare_88 (1.3e+06)
End 50kb
Mare_89 (1.3e+06)
Start 50kb
Mare_89 (1.3e+06)
End 50kb
Mare_92 (1.2e+06)
Start 50kb
Mare_92 (1.2e+06)
End 50kb
Mare_110 (1.0e+06)
Start 50kb
Mare_110 (1.0e+06)
End 50kb
Mare_128 (7.8e+05)
Start 50kb
Mare_128 (7.8e+05)
End 50kb
Mare_137 (7.1e+05)
Start 50kb
Mare_137 (7.1e+05)
End 50kb
Mare_148 (5.9e+05)
Start 50kb
Mare_148 (5.9e+05)
End 50kb
Mare_158 (4.8e+05)
Start 50kb
Mare_158 (4.8e+05)
End 50kb
Mare_170 (4.0e+05)
Start 50kb
Mare_170 (4.0e+05)
End 50kb
Mare_171 (4.0e+05)
Start 50kb
Mare_171 (4.0e+05)
End 50kb
Mare_176 (3.4e+05)
Start 50kb
Mare_176 (3.4e+05)
End 50kb
Mare_179 (3.2e+05)
Start 50kb
Mare_179 (3.2e+05)
End 50kb
Mare_196 (2.3e+05)
Start 50kb
Mare_196 (2.3e+05)
End 50kb
Mare_199 (2.1e+05)
Start 50kb
Mare_199 (2.1e+05)
End 50kb
Mare_201 (2.1e+05)
Start 50kb
Mare_201 (2.1e+05)
End 50kb

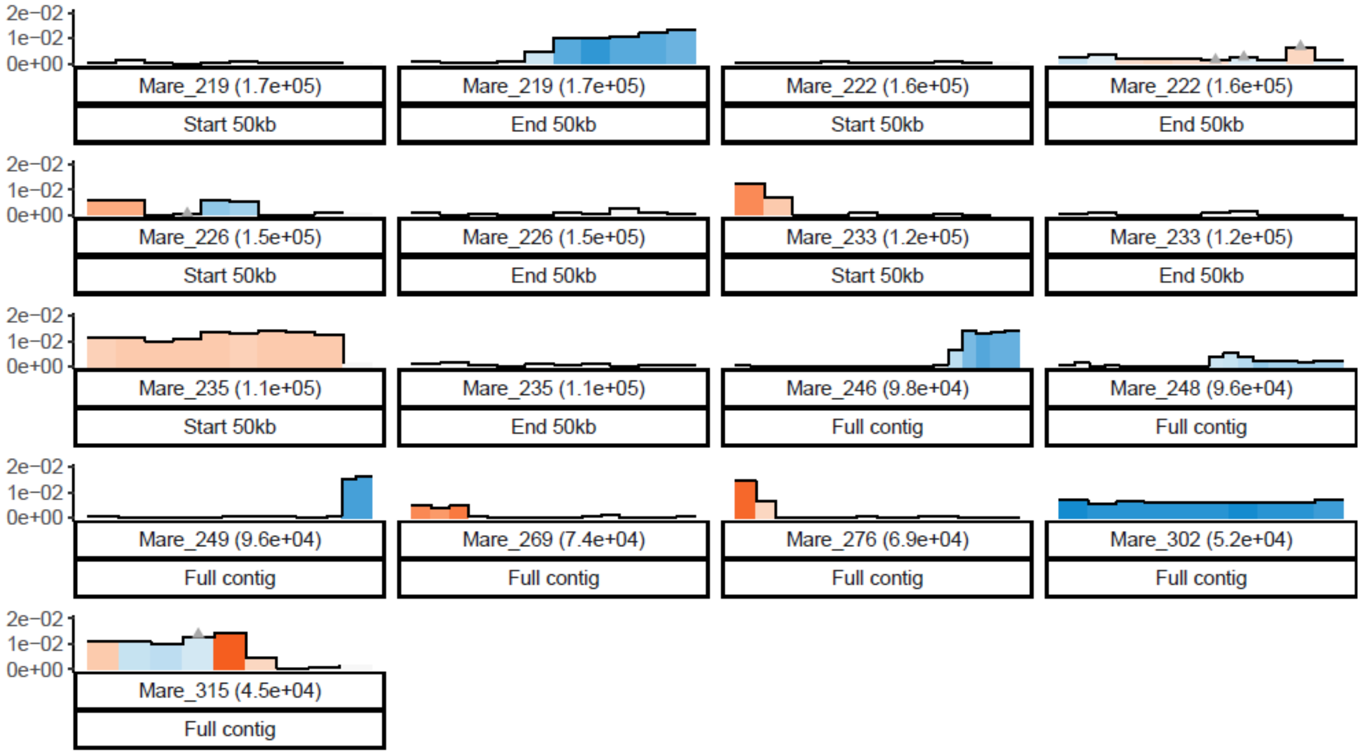

B) Density of repeat patterns and G4-quadruplex along *M. arenaria* contig extremities. The density of *Mare* repeat units is represented by a color gradient with positive values (red) indicating a density in the sense strand and negative values (blue) indicating a density on the reverse complement strand. Gray triangles above bars indicate regions of the contigs where less than 100% of the repeat patterns are on the same strand. The heights of bars in the histogram represent the density of G4-quadruplex forming regions. The first and last 50kb of each contig containing at least 3 repeats are represented and the values are per 5 kb windows on the genome, contigs that do not contain repeats in their first or last 50 kb are ignored. The distribution of Mare repeats and G4s on the whole contig length in 100 kb windows is available online at (https://doi.org/10.57745/CY06YE). Source data to produce this figure is provided as a Source Data file.

Supplementary Table 2: annotation of (retro)transposons and other repeats

The annotation of transposable elements and other repetitive regions on the genomes of *M. incognita*, *M. arenaria,* and *M. javanica* was performed using EDTA [8] version 2.1.0.

		M. incognita		*M. arenaria*		*M. javanica*	
Class	Type	Count	bpMasked	Count	bpMasked	Count	bpMasked
**Retrotransposons**							
LTR	Copia	25	8,783	444	185,968	290	134,146
	Gypsy	10216	5,548,981	12585	12,152,090	9704	8,940,100
	Unknown	11048	6,556,744	25891	11,396,311	11646	5,609,431
nonLTR	LINE_element	370	373,486	503	793,499	528	703,477
**DNA Transposons**							
TIR	CACTA	4296	896,156	5464	1,186,379	4370	1,113,443
	Mutator	30418	7,227,848	48371	12,631,831	54636	17,274,061
	PIF_Harbinger	2231	522,128	2513	696,,702	2096	531,815
	Tc1_Mariner	476	178,149	2128	522,,780	1963	524,585
	hAT	55062	11,913,136	94287	22,654,778	85261	19,036,926
	polinton	2561	2,988,992	1755	1,709,300	4784	5,536,271
nonTIR	helitron	51164	2,635,069	4369	4,726,375	4077	5,409,339
**Unclassified / unknown repeats**							
repeat region	repeat	27,789	8,705,800	45567	19,646,238	55511	19,620,486

Supplementary Figure 17. Gel electrophoresis of amplified telomere sequence.

Gradient PCR using primer pair and temperature range as listed in the Methods section in *M. incognita* (A), M. *javanica* (B), and *M. arenaria* (C). Labeled probe for telomere sequence using biotin-dUTP nucleotide in PCR reaction for *M. incognita* (D), and *M. javanica* as well as *M. arenaria* (E).

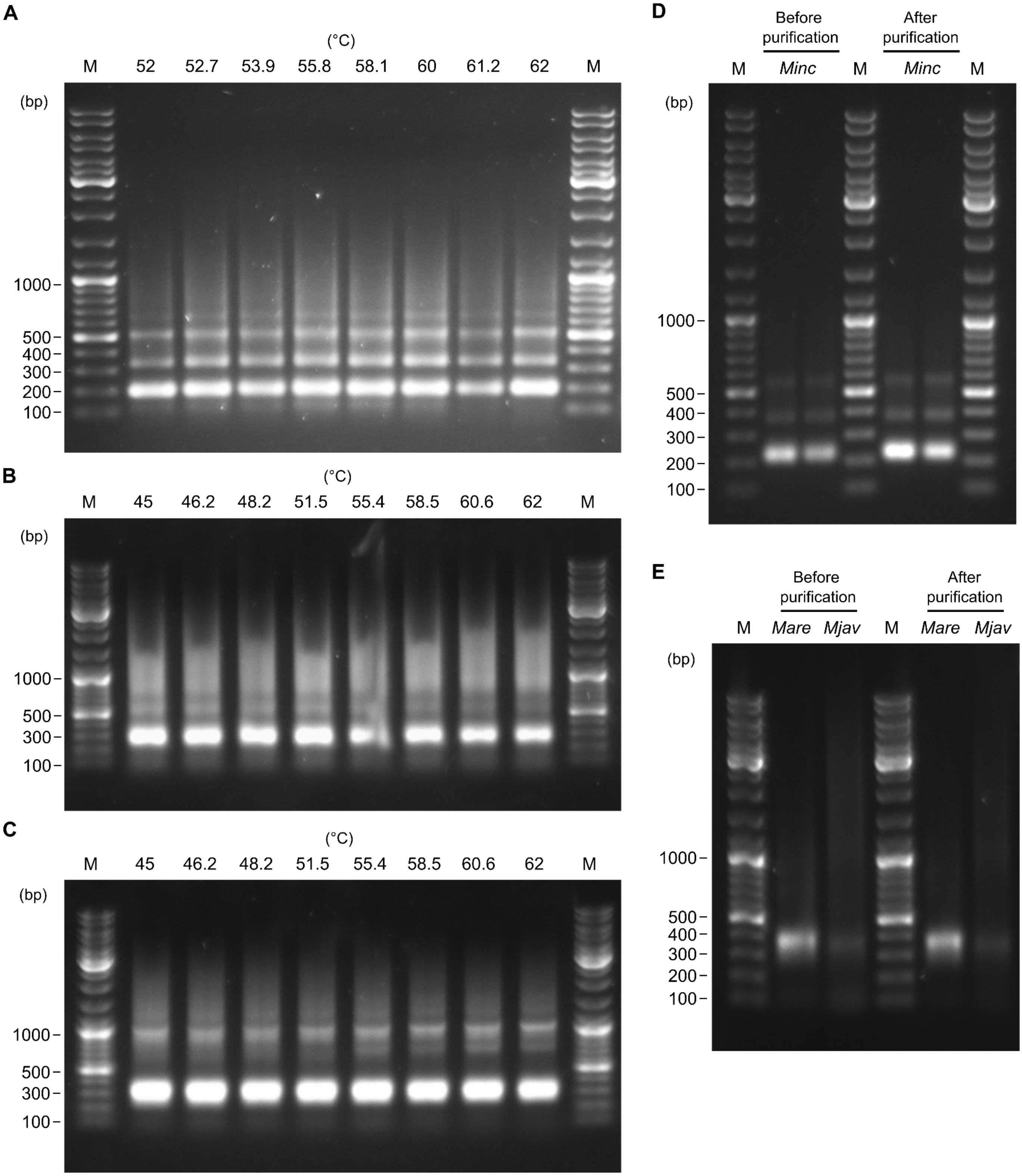

Supplementary Figure 18. FISH validation of telomeric localization of the repeat on *Minc* chromosomes

A high-resolution multi-panel image confirming specific FISH signal at telomeric position on *Minc* chromosomes as well as chromosomes and repeat arrays count in nuclei on several additional phases of the cell cycle https://doi.org/10.57745/5SXZQH

Supplementary Figure 19. Animated GIFs of FISH signal confirming specific localization at *Minc* chromosome extremities

Animated GIFs are available online at Recherche Data Gouv: https://doi.org/10.57745/OWZLRN with A) a 3D-reconstructed animation of *Minc* chromosomes with FISH signal and B) an animated GIF with an alternative display of the chromosome staining channel and fluorescence channel.

Supplementary Figure 20. Telomere localization in *M. javanica* (A) and *M. arenaria* (B).

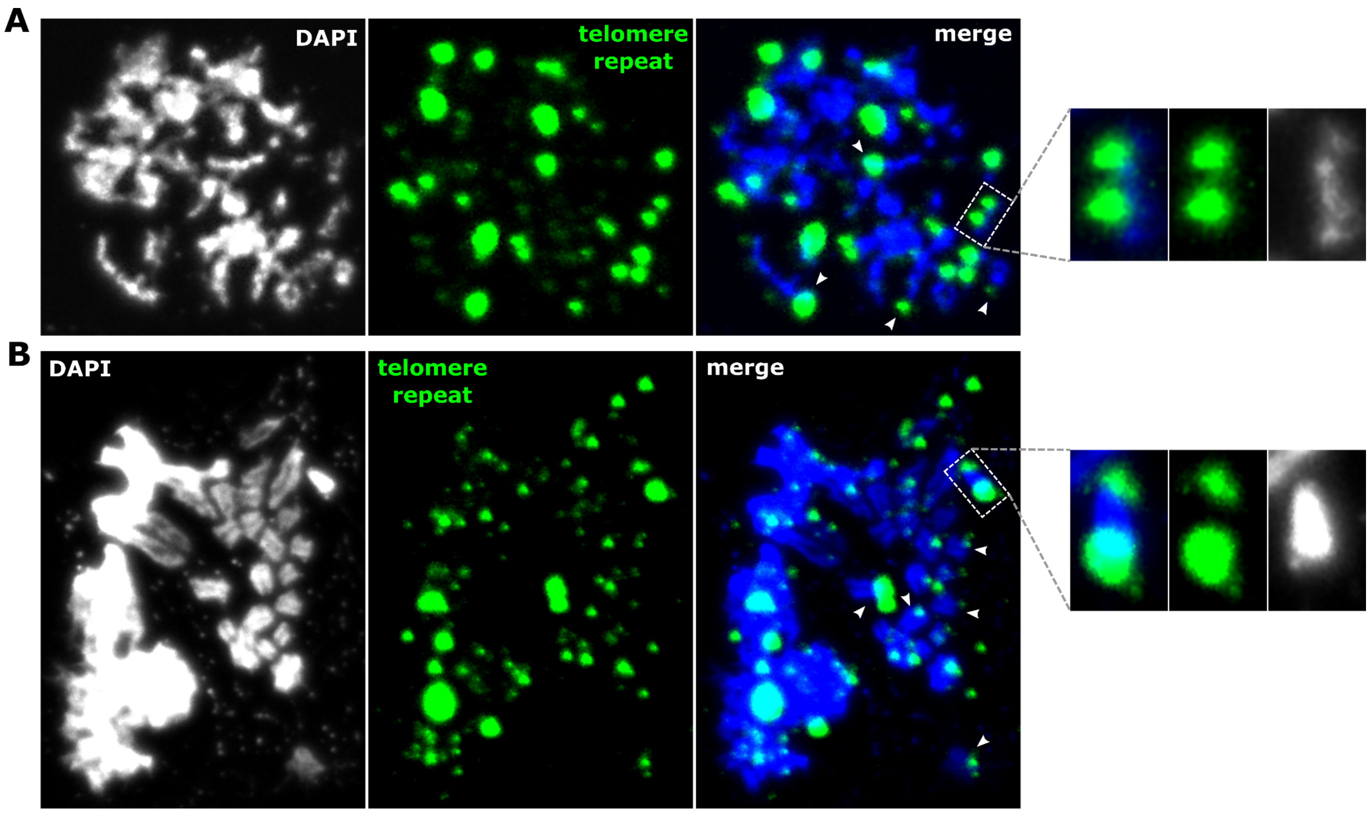

Chromosomes with visible telomere positions at their end are marked with white arrows. Enlarged on the right are chromosomes that show telomeric localization at both ends.

Supplementary Table 3: Raw Illumina data used for genome polishing and k-mer analyses

Species	Raw sequencing library	Reads	Length	Accession numbers
M. incognita	BZU_AAOSDE_1_1_H732LBCX2.12BA290_clean.fastq	19,805,212	250	ERX10638998
M. incognita	BZU_AAOSDE_1_2_H732LBCX2.12BA290_clean.fastq	19,805,212	250	ERX10638998
M. incognita	BZU_AAOSDE_2_1_H732LBCX2.12BA290_clean.fastq	20,090,514	250	ERX10638999
M. incognita	BZU_AAOSDE_2_2_H732LBCX2.12BA290_clean.fastq	20,090,514	250	ERX10638999
M. javanica	BZU_ACOSDE_2_1_H52YLBCX2.12BA292_clean.fastq	64,456,448	250	ERX10639000
M. javanica	BZU_ACOSDE_2_2_H52YLBCX2.12BA292_clean.fastq	64,456,448	250	ERX10639000
M. arenearia	BZU_ABOSDE_1_1_H732LBCX2.12BA291_clean.fastq	27,049,894	250	ERX10639001
M. arenearia	BZU_ABOSDE_1_2_H732LBCX2.12BA291_clean.fastq	27,049,894	250	ERX10639001
M. arenearia	BZU_ABOSDE_2_1_H732LBCX2.12BA291_clean.fastq	27,421,721	250	ERX10639002
M. arenearia	BZU_ABOSDE_2_2_H732LBCX2.12BA291_clean.fastq	27,421,721	250	ERX10639002

Supplementary Table 4: Primer sequences used in telomere FISH experiments

	Left primer	Right primer	Annealing temperature (°C)
M. incognita	TCGTCCATAGGTTCAGGCTT	GGGTCTACTTGGGTGGGG	62
M. javanica	AAGTTGTAGACGTCTGGCAGG	TCCTCTCCATAGGTTCAGGC	51.5
M. arenaria	GGGGTCATTAGAGTACCGTGTC	TTCAGGCTTTCATACCTCCGAG	55.4

References

1. Ranallo-Benavidez, T. R., Jaron, K. S. & Schatz, M. C. GenomeScope 2.0 and Smudgeplot for reference-free profiling of polyploid genomes. *Nat Commun* **11**, 1–10 (2020).
2. Mapleson, D., Garcia Accinelli, G., Kettleborough, G., Wright, J. & Clavijo, B. J. KAT: a K-mer analysis toolkit to quality control NGS datasets and genome assemblies. *Bioinformatics* **33**, 574–576 (2017).
3. Blanc-Mathieu, R. *et al.* Hybridization and polyploidy enable genomic plasticity without sex in the most devastating plant-parasitic nematodes. *PLOS Genetics* **13**, e1006777 (2017).
4. Sallet, E., Gouzy, J. & Schiex, T. EuGene: An Automated Integrative Gene Finder for Eukaryotes and Prokaryotes. *Methods Mol. Biol.* **1962**, 97–120 (2019).
5. Girgis, H. Z. Red: an intelligent, rapid, accurate tool for detecting repeats de-novo on the genomic scale. *BMC Bioinformatics* **16**, 227 (2015).
6. Maddison, W. P. & Maddison, D. R. Mesquite: A modular system for evolutionary analysis. Version 3.01. (2014).
7. Yoshida, Y. *et al.* Comparative genomics of the tardigrades Hypsibius dujardini and Ramazzottius varieornatus. *PLOS Biology* **15**, e2002266 (2017).
8. Ou, S. *et al.* Benchmarking transposable element annotation methods for creation of a streamlined, comprehensive pipeline. *Genome Biology* **20**, 275 (2019).
