# Supplementary material for: Unzipped genome assemblies of polyploid root-knot nematodes reveal unusual and clade-specific telomeric repeats
Source: Nat Commun. 2024 Feb 5;15:773. doi: 10.1038/s41467-024-44914-y (PMC10844300; doi:10.1038/s41467-024-44914-y)
Supplement: Supplementary file 3 — Descrition of Supplementary Data Files [file 41467_2024_44914_MOESM3_ESM.pdf]

Description of Additional Supplementary Files

Supplementary Data 1: Distribution of (TTAGGC)n repeat and telomere-associated proteins across nematode genomes.

Sources of genomes and predicted proteomes as well taxonomic classification are provided. - First sheet: Full details of the source for genomes and proteomes and evidence for presence / absence of the TTAGGC repeat and telomere-associated proteins. - Second sheet: Consensus final decision for presence / absence of the TTAGGC repeat and telomere-associated proteins.

Supplementary Data 2: Positions of *M. incognita* repeats on the contigs.

Results of a BLASTn search with the *M. incognita* repeat as a query against the *M. incognita* contigs with an e-value threshold of 1e-35. Positions of the composite repeats, their orientation and presence / absence of the hmm profiles for the 3 constitutive motifs are indicated as well as the assignment to either an A or B subgenome according to McScanX analysis.

Supplementary Data 3: Positions of *M. javanica* repeats on the contigs.

Results of a BLASTn search with the *M. javanica* repeat as a query against the *M. javanica* contigs with an e-value threshold of 1e-35. Positions of the composite repeats, their orientation and presence / absence of the hmm profiles of the 3 constitutive motifs are indicated as well as the assignment to either an A or B subgenome according to McScanX KS analysis.

Supplementary Data 4: Positions of *M. arenaria* repeats on the contigs.

Results of a BLASTn search with the *M. arenaria* repeat as a query against the *M. arenaria* contigs with an e-value threshold of 1e-35. Positions of the composite repeats, their orientation and presence / absence of the hmm profiles of the 3 constitutive motifs are indicated as well as the assignment to either an A or B subgenome according to McScanX KS analysis.

Supplementary Data 5: Ratio between repetitive elements and coding regions.

Analysis of all contigs from Minc, Mjav and Mare. For each species, we counted the number of nucleotides in both coding (CDS) and Repetitive regions at the ends of all contigs. We calculated the ratio of these bases (RE/CDS) in order to define a threshold for contig extremities containing telomeres. We then checked all the contigs with a high ratio, for identification of other putative composite repeats.

Supplementary Data 6: Clustering of motif-1 occurrences and cross-reference with A/B subgenomes assignment.

All motif-1 occurrences of the 3 species (Minc, Mjav, Mare) were clustered at 99.9% identity using CD-HIT and clustering results were cross-referenced with A/B subgenomes assignments. Sheet 1: The 52 clusters with information of the species and contig of origin as well as positions on the contigs. Sheet 2: Distribution of Minc motif-1 occurrences across the clusters with information of A/B subgenome of origin. Sheet 3: Sheet 2: Distribution of Mare motif-1 occurrences across the clusters with information of A/B subgenome of origin. Sheet 4: Distribution of Mjav motif-1 occurrences across the clusters with information of A/B subgenome of origin. Sheet 5: Cross-species motif-1 occurrences across the clusters with information of A/B subgenome of origin. Sheet 6: Distribution of species per cluster.

Supplementary Data 7: Positions of Minc repeats on Mluci contigs.

Positions of Minc repeats on Mluci contigs. Results of a BLASTn search with the M. incognita repeat as a query against the M. luci contigs. Positions of the composite repeats, their orientation and presence / absence of the 3 constitutive motifs are indicated.

Supplementary Data 8: *de novo* assembled Minc transcripts containing the repeat and matching the genome.

We assembled the transcriptomes of four *M. incognita* developmental life stages RNA-seq datasets using TRINITY. W= egg, J2= pre-parasitic second stage juveniles, J3= a mix of J3 and J4 endophytic stages, F= adult female. We searched for the presence of the motifs constituting the Minc telomeric repeat. Transcripts that contained at least one of the 3 Minc telomeric motifs were mapped to the genome using GMAP.

Supplementary Data 9: ISO-seq Minc transcripts containing the repeat and matching the genome.

We used the publicly available data of IsoSeq sequencing of *Meloidogyne incognita* (available under NCBI accessions PRJNA787737, SRP350177. The data were obtained using a mix of *M. incognita* developmental stages of. We searched for the presence of telomeric motifs in the ISO-seq reads. We mapped the motif-containing ISO-seq reads to the reference genome of *M. incognita* using GMAP with default parameters. A summary of the mapping results is provided here.

Supplementary Data 10: de novo assembled Mjav transcripts containing the repeat and matching the genome.

We assembled the transcriptomes of *M. javanica* J2 life stage RNA-seq datasets using TRINITY. We searched for the presence of the motifs constituting the Mjav telomeric repeat on the assembled transcripts using blastn (Sheet1). We then selected the transcripts that contained at least one of the 3 Mjav telomeric motifs mapped to the genome using GMAP (Sheet2).

Supplementary Data 11: de novo assembled Mare transcripts containing the repeat and matching the genome.

We assembled the transcriptomes of *M. arenaria* J2 life stage RNA-seq datasets using TRINITY. We searched for the presence of the motifs constituting the Mare telomeric repeat on the assembled transcripts using blastn (Sheet1). We then selected the transcripts that contained at least one of the 3 Mare telomeric motifs mapped to the genome using GMAP (Sheet2).

Supplementary Data 12: Information on nanopore sequencing libraries.

Sequencing libraries were produced using the Minion or Promethion platform, with flow-cell version 9.4.1 in both cases. Libraries sequenced with a Minion device were then base-called using guppy dna_r9.4.1_450bps_sup.cfg configuration file. Libraries sequenced with a Promethion device were then base-called using guppy dna_r9.4.1_450bps_sup_prom.cfg configuration file. All base calling was made in super high accuracy mode.
