# Supplementary material for: Unzipped genome assemblies of polyploid root-knot nematodes reveal unusual and clade-specific telomeric repeats
Source: Nat Commun. 2024 Feb 5;15:773. doi: 10.1038/s41467-024-44914-y (PMC10844300; doi:10.1038/s41467-024-44914-y)
Supplement: Supplementary file 16 — Reporting Summary [file 41467_2024_44914_MOESM16_ESM.pdf]

nature portfolio

Corresponding author(s): Etienne GJ Danchin

Last updated by author(s): Dec 18, 2023

Reporting Summary

Nature Portfolio wishes to improve the reproducibility of the work that we publish. This form provides structure for consistency and transparency in reporting. For further information on Nature Portfolio policies, see our Editorial Policies and the Editorial Policy Checklist.

Statistics

For all statistical analyses, confirm that the following items are present in the figure legend, table legend, main text, or Methods section.

n/a	Confirmed	
☒	☐	The exact sample size (*n*) for each experimental group/condition, given as a discrete number and unit of measurement
☒	☐	A statement on whether measurements were taken from distinct samples or whether the same sample was measured repeatedly
☐	☒	The statistical test(s) used AND whether they are one- or two-sided<br>*Only common tests should be described solely by name; describe more complex techniques in the Methods section.*
☒	☐	A description of all covariates tested
☒	☐	A description of any assumptions or corrections, such as tests of normality and adjustment for multiple comparisons
☐	☒	A full description of the statistical parameters including central tendency (e.g. means) or other basic estimates (e.g. regression coefficient) AND variation (e.g. standard deviation) or associated estimates of uncertainty (e.g. confidence intervals)
☒	☐	For null hypothesis testing, the test statistic (e.g. *F*, *t*, *r*) with confidence intervals, effect sizes, degrees of freedom and *P* value noted<br>*Give P values as exact values whenever suitable.*
☒	☐	For Bayesian analysis, information on the choice of priors and Markov chain Monte Carlo settings
☒	☐	For hierarchical and complex designs, identification of the appropriate level for tests and full reporting of outcomes
☒	☐	Estimates of effect sizes (e.g. Cohen's *d*, Pearson's *r*), indicating how they were calculated

Our web collection on statistics for biologists contains articles on many of the points above.

Software and code

Policy information about availability of computer code

Data collection	No software was used for data collection
Data analysis	ONT reads high accuracy basecalling: Guppy (v6.0.6). ONT reads quality check: cONTent (https://github.com/DjampaKozlowski/cONTent). ONT reads filtering: Nanofilt. Genomes assemblies: Necat (version 20200119). Genomes polishing with long reads: Racon (version 1.4.10 and Medaka (version 1.4.4). Genomes polishing with short reads: Hapo-G (version 1.2). K-mer analyses: JellyFish (v. 2.3.0), GenomeScope2 and Smudgeplot. Recovery of universally conserved genes: CEGMA (v2.5) and BUSCO (v5.4.4). Telomeric repeat search: TidK, MEME suite, EMBOSS suite, BLAST, HMMER. Orthology search: OrthoFinder, BLAST, Diamond. Gene predictions: EuGene. Duplications detection and classification: McScanX. Repeat and transposable elements predictions: EDTA. G-quadruplex search: G4-hunter. Transcriptomes assemblies: Trinity. Alignments of transcripts on genomes: GMAP.<br>Custom / unpublished code: All the custom codes and scripts used in this study are publicly available on GitHub without restriction for re-use with links to the resources cited at the appropriate position in the manuscript. All already published codes are cited by their references. cONTent [https://github.com/DjampaKozlowski/cONTent] (ONT reads quality and length distribution quality check). Medaka [https://github.com/nanoporetech/medaka] (ONT polish software). Collinearity [https://github.com/reubwn/collinearity/tree/master] (ka/ks result from MCscanX formatting). AssignGenomes [https://github.com/azotta/MeloidogyneGenomes] (Assign the A and B genomes of Meloidogyne). TIDK [https://github.com/tolkit/telomeric-identifier] (Canonical Telomeric sequence identification).

For manuscripts utilizing custom algorithms or software that are central to the research but not yet described in published literature, software must be made available to editors and reviewers. We strongly encourage code deposition in a community repository (e.g. GitHub). See the Nature Portfolio guidelines for submitting code & software for further information.

Data

Policy information about availability of data

All manuscripts must include a data availability statement. This statement should provide the following information, where applicable:

- Accession codes, unique identifiers, or web links for publicly available datasets
- A description of any restrictions on data availability
- For clinical datasets or third party data, please ensure that the statement adheres to our policy

All the raw long and short genome sequencing reads for the three species have been deposited in the EBI's European Nucleotide Archive (ENA) under BioProject PRJEB61149 [https://www.ncbi.nlm.nih.gov/bioproject/PRJEB61149] with detail of each library and accession numbers in Supplementary Data 12 and Table 3. RNA-seq raw data used for transcriptome assembly of Minc, Mjav, and Mare are under BioProject PRJEB8846, [https://www.ncbi.nlm.nih.gov/bioproject/PRJEB8846] (Minc RNA-Seq raw data), BioProject PRJEB8843, [https://www.ncbi.nlm.nih.gov/bioproject/PRJEB8843] (Mjav RNA-Seq raw data), and BioProject PRJEB8845, [https://www.ncbi.nlm.nih.gov/bioproject/PRJEB8845] (Mare RNA-Seq raw data). Genome and transcriptome assemblies as well as annotations have been deposited in the French national data repository 'Recherche Data Gouv' and are publicly accessible at this address: https://entrepot.recherche.data.gouv.fr/dataverse/Melo-Telo/. The ISO-seq data was retrieved from BioProject PRJNA787737 [https://www.ncbi.nlm.nih.gov/sra/PRJNA787737]. The source data for Figures 1, and 3 and Supplementary Figures 14, 15, and 16 are provided as a Source Data file.
Sources for genomes and predicted proteins: WormBase ParaSite, NCBI Genome Resource (details in Supplementary Data 1). RNA-seq data: EMBL-EBI European Nucleotide Archive (ENA): ERA419974. ISO-seq data: NCBI (PRJNA787737 SRP350177). C. elegans telomerase and other telomere-related proteins: WormBase. Protein motifs and domains: PANTHER, Pfam, InterPro.

Research involving human participants, their data, or biological material

Policy information about studies with human participants or human data. See also policy information about sex, gender (identity/presentation), and sexual orientation and race, ethnicity and racism.

Reporting on sex and gender	This research does not involve human participants, their data or biological material
Reporting on race, ethnicity, or other socially relevant groupings	This research does not involve human participants, their data or biological material
Population characteristics	*Describe the covariate-relevant population characteristics of the human research participants (e.g. age, genotypic information, past and current diagnosis and treatment categories). If you filled out the behavioural & social sciences study design questions and have nothing to add here, write "See above."*
Recruitment	*Describe how participants were recruited. Outline any potential self-selection bias or other biases that may be present and how these are likely to impact results.*
Ethics oversight	*Identify the organization(s) that approved the study protocol.*

Note that full information on the approval of the study protocol must also be provided in the manuscript.

Field-specific reporting

Please select the one below that is the best fit for your research. If you are not sure, read the appropriate sections before making your selection.

☒ Life sciences ☐ Behavioural & social sciences ☐ Ecological, evolutionary & environmental sciences

For a reference copy of the document with all sections, see nature.com/documents/nr-reporting-summary-flat.pdf

Life sciences study design

All studies must disclose on these points even when the disclosure is negative.

Sample size	The strains have been maintained in our live nematode collection for 30 years on tomato. For each Meloidogyne species sequenced (M. incognita, M. javanica and M. arenaria), and for each sequencing run, ca. 150,000 eggs were collected to produce enough DNA for genome sequencing. The eggs were collected 2 months after inoculation on tomato roots. No statistics were produced to determine sample size, we knew from our previous sequencing experiments that this sample size produced a sufficient quantity of DNA compatible with long read genome sequencing.
Data exclusions	No data was excluded in this study
Replication	Transcriptome data was solely used to identify evidence for transcription either to predict genes or to predict TERRA. No differential gene expression analysis was performed in this study. For trancriptome data of each developmental life stage in M. incognita, three biological replicates had been produced as part of another publication (Blanc-Mathieu et al. 2017, ref. 20 in the manuscript). All attempts of replications were succesful as shown in the previous publication (Blanc-Mathieu et al. 2017, ref. 20 in the manuscript).
Randomization	There was no experimental groups in this study as no comparison between conditions were done.

Blinding | Blinding was not relevant for this study because no factor related to sex or age or other factor could influence study results.

Reporting for specific materials, systems and methods

We require information from authors about some types of materials, experimental systems and methods used in many studies. Here, indicate whether each material, system or method listed is relevant to your study. If you are not sure if a list item applies to your research, read the appropriate section before selecting a response.

Materials & experimental systems

n/a	Involved in the study
☒	☐ Antibodies
☒	☐ Eukaryotic cell lines
☒	☐ Palaeontology and archaeology
☐	☒ Animals and other organisms
☒	☐ Clinical data
☒	☐ Dual use research of concern
☐	☒ Plants

Methods

n/a	Involved in the study
☒	☐ ChIP-seq
☒	☐ Flow cytometry
☒	☐ MRI-based neuroimaging

Animals and other research organisms

Policy information about studies involving animals; ARRIVE guidelines recommended for reporting animal research, and Sex and Gender in Research

Laboratory animals	For each Meloidogyne (root-knot nematode) species, we have sequenced clones from the progeny of one original single female. Eggs from Meloidogyne incognita strain Morelos, Meloidogyne javanica strain Avignon and Meloidogyne arenaria strain Guadeloupe (from INRAE Sophia Antipolis collection) were collected, 6 weeks after inoculation, from tomato roots (Solanum esculentum cv. St Pierre). Before storage at -80°C, eggs were purified from root debris by sucrose gradient centrifugations.
Wild animals	No wild animals were used in this study, only nematodes from INRAE collection and multiplied in our laboratory.
Reporting on sex	These species are parthenogenetic with only females participating to the offspring. We sequenced the eggs which are sexually undifferentiated.
Field-collected samples	We have sequenced clones from the progeny of one original single female that were collected, 6 weeks after inoculation, from tomato roots (Solanum esculentum cv. St Pierre).
Ethics oversight	No ethical approval or guidance was required for this study as it concerns genome analysis of organisms reared in an INRAE nematode collection.

Note that full information on the approval of the study protocol must also be provided in the manuscript.

Dual use research of concern

Policy information about dual use research of concern

Hazards

Could the accidental, deliberate or reckless misuse of agents or technologies generated in the work, or the application of information presented in the manuscript, pose a threat to:

No	Yes
☒	☐ Public health
☒	☐ National security
☒	☐ Crops and/or livestock
☒	☐ Ecosystems
☒	☐ Any other significant area

Experiments of concern

Does the work involve any of these experiments of concern:

No	Yes	
☒	☐	Demonstrate how to render a vaccine ineffective
☒	☐	Confer resistance to therapeutically useful antibiotics or antiviral agents
☒	☐	Enhance the virulence of a pathogen or render a nonpathogen virulent
☒	☐	Increase transmissibility of a pathogen
☒	☐	Alter the host range of a pathogen
☒	☐	Enable evasion of diagnostic/detection modalities
☒	☐	Enable the weaponization of a biological agent or toxin
☒	☐	Any other potentially harmful combination of experiments and agents
